# Synchronous and Asynchronous Variation of Taxonomic and Phylogenetic Diversity During the Succession of *Pinus kesiya* var. *langbianensis* Forest in Yunnan, China

**DOI:** 10.1002/ece3.72911

**Published:** 2026-01-12

**Authors:** Xiaofan Wang, Yunfei Ma, Biao Zhao, Dengpeng Chen, Yehong Luo, Mingchun Peng, Yongping Li, Xinmao Zhou, Wen Chen, Cindy Q. Tang, Chongyun Wang

**Affiliations:** ^1^ State Key Laboratory of Vegetation Structure, Function and Construction (VegLab) Yunnan University Kunming China; ^2^ Institute of Ecology and Geobotany, School of Ecology and Environmental Science Yunnan University Kunming China; ^3^ Southwest United Graduate School Yunnan University Kunming China; ^4^ School of Agriculture Yunnan University Kunming China

**Keywords:** community assembly, *Pinus kesiya* var. *langbianensis*, space‐for‐time‐substitution, succession, α diversity, β diversity

## Abstract

The changing patterns of taxonomic diversity (TD) and phylogenetic diversity (PD) during forest succession can provide a reference for optimizing forest ecosystem management. The widely distributed *Pinus kesiya* var. *langbianensis* forest (PKF) in subtropical Yunnan, China, has important ecological and economic values. However, little is known about species diversity patterns and driving factors during the pine forest succession. Adopting the “space‐for‐time‐substitution” (SFTS) approach and on the basis of community data from different successional stages, we investigated the dynamics of TD and PD across PKF succession by integrating environmental and spatial variables. The results show that both TD and PD follow a cosine pattern during succession, peaking at mid‐ to late‐successional stages, but with TD lagging behind PD. TD responds more rapidly to changes in dominant environmental factors than PD. Moreover, there is an asynchronous oscillation between taxonomic β diversity (TβD) and phylogenetic β diversity (PβD). TβD is consistently greater than PβD and increases monotonically throughout succession, whereas the PβD still fluctuates in a cosine pattern. TD and PD are equally important in maintaining community stability, and the community becomes increasingly homogeneous and stable. Notably, early and late successional stages are dominated by competitive exclusion, whereas environmental filtering prevailed at mid‐succession. The mean temperature of the driest quarter (bio9) plays an environmental filtering role in the community composition at the ecological scale, and the precipitation of the coldest quarter (bio19) shapes the phylogenetic structure by influencing the regional species pool at the evolutionary scale. Neutral and deterministic processes jointly govern β diversity, but niche differentiation has an increasing domination, which supports the “successional continuum hypothesis”. Spatial effects must be explicitly considered in SFTS‐based successional studies. The management should prioritize conserving mid‐successional stages (peak diversity) and balancing environmental heterogeneity with dispersal limitation. Ecological‐evolutionary assembly linkages should be considered in the pinewood sustainable utilization and management.

## Graphical abstract

Based on community data and the space‐for‐time‐substitution (SFTS) approach, this study shows that both taxonomic and phylogenetic diversity follow a cosine pattern during succession, with PD peaking earlier than TD. Community stability increases as niche differentiation strengthens, with competitive exclusion dominating early and late stages, and environmental filtering prevailing mid‐succession. Mid‐successional stages are critical for biodiversity conservation and should be prioritized in forest management.

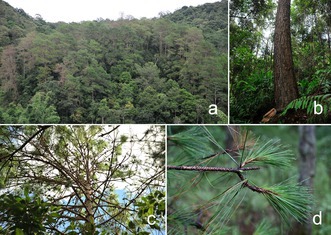

## 1 Introduction

In the 18th century, European foresters first discussed the concept of forest succession (Spurr 1952). Succession has remained a central theme in ecology for more than a century (Walker and Del Moral 2003; Prach and Walker 2011; Letten et al. 2014; Meiners et al. 2015; Martínez‐Ramos et al. 2021; Poorter et al. 2024). Vegetation succession is a dynamic process of community assembly (Norden et al. 2009; Lebrija‐Trejos et al. 2010; Omidipour et al. 2021). As succession progresses, intra‐ or interspecific interactions intensify, the light‐use efficiency within the community increases, and resource allocation shifts. Consequently, community assembly patterns become increasingly heterogeneous among communities (Lohbeck et al. 2013, 2014; Purschke et al. 2013).

Two traditional theories have been proposed to explain community assembly during succession: the niche theory (Diamond 1975; Webb 2000; Webb et al. 2002) and the neutral theory (Hubbell 2011). The niche theory holds that community assembly is dominated by deterministic processes, such as environmental filtering or competitive exclusion (Chave 2004), whereas the neutral theory holds that community assembly is controlled by dispersal limitation or stochastic processes (Hubbell 2011). However, many researchers have increasingly recognized that these two processes coexist and complement each other in community assembly dynamics (Niu et al. 2009; Poorter et al. 2010), as their relative importance shifts across successional stages (Purschke et al. 2013; Lohbeck et al. 2014). There is still uncertainty in community assembly processes during succession. For example, the secondary and primary forests exhibit distinct assembly mechanisms at each stage (Bhaskar et al. 2014). The community assembly pattern of secondary forests is deterministic to a great extent, driven by the species' life history strategies and interspecific competition (Norden et al. 2012; Zhang et al. 2014; Lanta et al. 2023). In tropical forests, environmental filtering predominates (Whitfeld et al. 2012), whereas in subtropical forests, deterministic forces exert a weaker influence during succession (Mi et al. 2016).

Vegetation dynamics are accurately revealed by long‐term plot studies (Warming 1895; Lasky et al. 2014) and can also provide insights into continuous processes that cannot be captured by the space‐for‐time substitution (SFTS) approach (Smale et al. 2016; Fickert and Grüninger 2018). However, there are many challenges for conventional experiments and observations, requiring time frames well beyond instant results (Fukami and Wardle 2005; Johnson and Miyanishi 2008). The SFTS approach not only requires fewer human, material, and financial resources than the long‐term plot method but also produces reasonably reliable results (Li et al. 2016). It is appropriate for studying convergent secondary succession with low biodiversity, rapid species turnover, and infrequent, low‐intensity disturbances (Walker et al. 2010). As a result, the SFTS approach is widely used to study succession by many ecologists (Cowles 1899; Whittaker 1975; Pickett 1989; Blois et al. 2013; Song et al. 2019; Romanowski et al. 2021; Kharouba and Williams 2024; van der Sande et al. 2024). Then, will the two methods lead to significant differences in species diversity patterns in the process of succession? Subsequently, numerous studies have evaluated the reliability and applicability of the SFTS method by comparing it with long‐term monitoring. For example, Foster and Tilman (2000) demonstrated that SFTS‐derived data closely predicted temporal changes in species abundance, with discrepancies largely attributable to extreme drought events. Similarly, SFTS predictions of plant compositional changes in eastern North America were consistent with 72% of those derived from long‐term plots (Miao et al. 2018). These findings collectively highlight both the extensive use and practical validation of SFTS. More recently, the method has been widely applied to infer temporal dynamics in both taxonomic and phylogenetic diversity within communities (Li et al. 2015; Liu et al. 2016; Geng et al. 2022; Fu et al. 2023; Yuan et al. 2023).

Forest community structure and biodiversity exhibit uncertainty in succession (Howard and Lee 2003; Yuan et al. 2011; Marcilio‐Silva et al. 2016; Chai et al. 2023). Taxonomic diversity (TD) and phylogenetic diversity (PD) complement each other to provide an enhanced perspective of community assembly, which is driven by deterministic or stochastic processes in succession (Norden et al. 2009; Pausas and Verdu 2010; Letcher et al. 2012; Purschke et al. 2013; Jin et al. 2015; Jarzyna and Jetz 2016; Liu et al. 2016), as well as evolutionary or ecological variation (Webb et al. 2002; Cavender‐Bares et al. 2009; Norden et al. 2012). However, TD and PD show different patterns of change during succession (Purschke et al. 2013, 2017; Vellend et al. 2013; Chai et al. 2016; Yu et al. 2019), and have different effects on ecosystem functioning (Ouyang et al. 2016; Yuan et al. 2016). This suggests that they may capture different ecological processes. Meanwhile, phylogenetic community structure changes from clustering to overdispersion or randomization from early to late succession, or shows an opposite pattern (Letcher 2010; Norden et al. 2012; Stadler et al. 2017; Yu et al. 2019; Mastrogianni et al. 2024). TD exhibits uncertainty during succession, and four main patterns can be distinguished: TD can monotonically decrease or increase with ongoing succession, peak at middle successional stages, or may not show any temporal trend (Odum 1983; Howard and Lee 2003; Tilman et al. 2006; Baniya et al. 2009; Rolo et al. 2016). PD may increase significantly with the trajectory of community succession (Marcilio‐Silva et al. 2016; Muscarella et al. 2016; McKone et al. 2021; Karimi et al. 2022); decrease (Verdú et al. 2009; Li et al. 2015; Chai et al. 2016; Lazzaro et al. 2020; Kong et al. 2023); or show no significant trend over time (Bruelheide et al. 2011; Norden et al. 2012; Mi et al. 2016; Xu et al. 2020; Chai et al. 2023). Therefore, it is particularly noteworthy that studies on TD and PD patterns during succession have yielded a series of puzzling and contradictory conclusions. Such discrepancies may stem from differences in regional climate and biogeography (Chai et al. 2016), forest types (Bruelheide et al. 2011; Norden et al. 2012), types of succession (Muscarella et al. 2016), the completeness of coverage along the successional continuum (Karimi et al. 2022), and the scale effects associated with different research approaches (Miao et al. 2018). At present, there is still a lack of systematic studies comparing the patterns of TD and PD within the same successional framework. A study that jointly examines TD and PD across a full successional gradient while accounting for spatial effects in the SFTS framework is essential for reconciling these contradictory patterns and clarifying the mechanisms of community assembly.

The significance of spatial ecological structure has been widely acknowledged (Legendre 1993). Because ecological data are inherently collected across geographic space, spatial relationships among sampling sites often lead to spatial dependence in community patterns (Borcard et al. 2004). Although spatial eigenvector‐based approaches have been widely applied to detect spatial structures across ecological systems, including ferns in Amazonia (Borcard et al. 2004), benthic algae (Wu et al. 2021), and fish diversity in floodplain lakes (Virgilio et al. 2022), their role in succession studies remains underappreciated. Classical SFTS‐based succession studies (Parrish and Bazzaz 1982; Yuan et al. 2023) implicitly assume that spatial processes are negligible, leading to an underestimation of dispersal limitation and spatial structuring. Therefore, explicitly incorporating spatial factors is essential for accurately interpreting successional dynamics and avoiding misattribution of spatial patterns to temporal processes.

**FIGURE 1:**
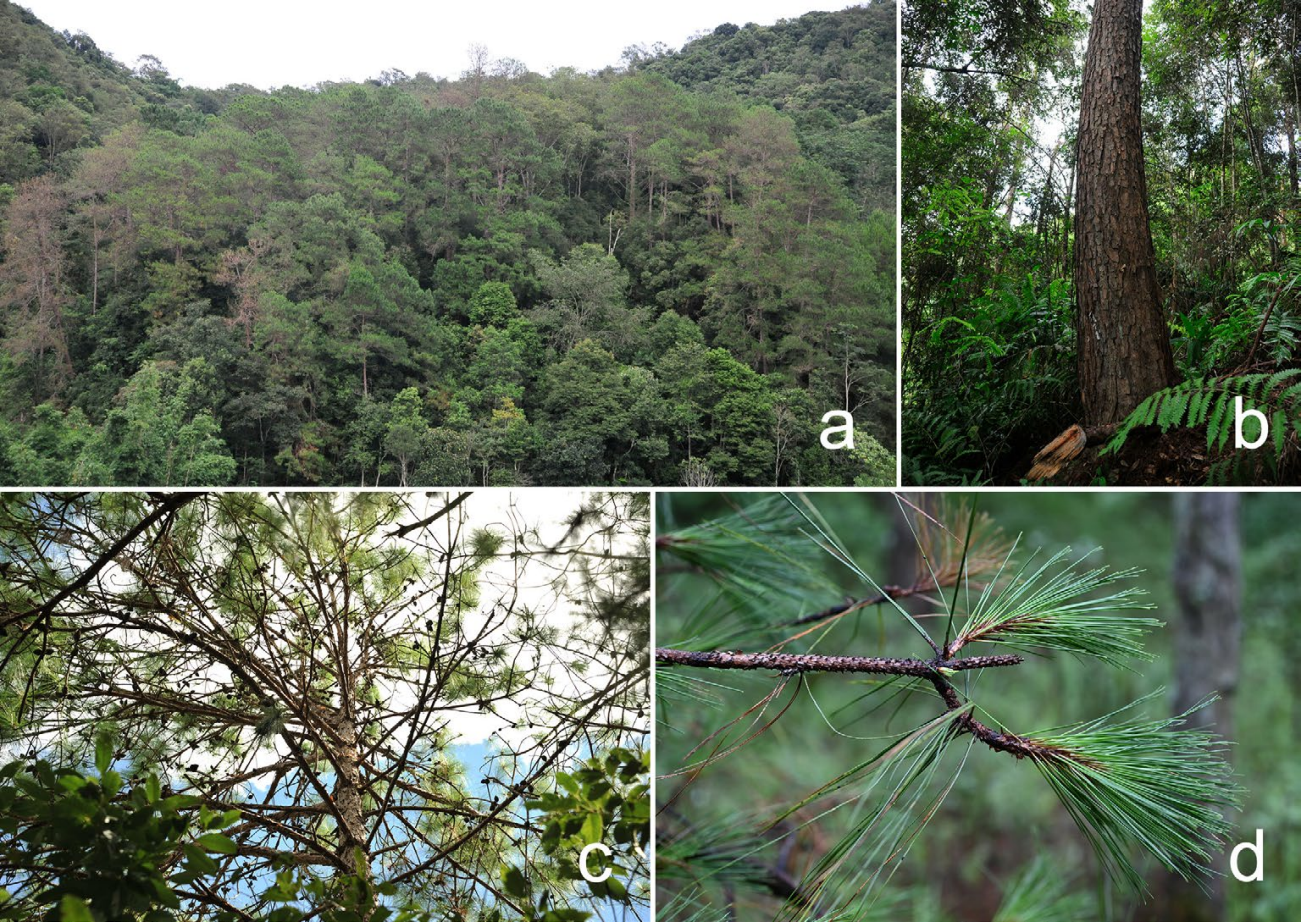
Structure and morphological characteristics of *P. kesiya* var. *langbianensis* forest (PKF) across the study area (Photographed by C. Y. Wang). (a) Community appearance; (b) trunk and understory vegetation; (c) crown structure; (d) branches and needles.

Forest succession is a fundamental process shaping terrestrial biodiversity worldwide (Crouzeilles et al. 2016). As one of the largest genera of conifers, pine (*Pinus*) forests are distributed across the entire Northern Hemisphere (Eckert and Hall 2006) and play a key role as pioneer communities in post‐disturbance landscapes (Sousa 1984). In particular, subtropical and tropical pine forests exhibit relatively rapid successional turnover, making them ideal model systems for investigating successional dynamics within observable time scales (Stephenson and van Mantgem 2005). *Pinus kesiya* var. *langbianensis*, Simao pine, is a geographical variant of *P. kesiya* Royle ex Gordon. Paleontological and molecular biological evidence support that this species may have differentiated southward from *Pinus prekesiya* with the continuous uplift of the Qinghai‐Tibet Plateau in the Neogene and the gradual strengthening of the South Asian monsoon climate (Xing et al. 2010). *P. kesiya* var. *langbianensis* is valuable for timber, pulp, and turpentine; more than 80% of turpentine production comes from it in Yunnan (Wu and Dang 1992). It is one of the typical pioneer tree species, spreading by winged seeds. Normally, *P. kesiya* var. *langbianensis* forest (PKF, Figure 1) will be replaced by monsoon evergreen broad‐leaved forests (Song et al. 2011). PKF is mainly distributed in the south subtropical and tropical lowland areas to the west of Ailao Mountain in Yunnan, China (Wu and Zhu 1987; Wen et al. 2010). As a typical secondary but long‐lasting forest, PKF stands are 1.48 × 10^6^ hm^2^, accounting for 6.5% of the forested area in Yunnan, and have a standing stock of about 1.50 × 10^9^ m^3^, giving it significant value for ecosystem service and carbon sequence (Wen et al. 2010).

Furthermore, PKF represents a transitional forest type, shifting from a relatively homogeneous coniferous community to a more heterogeneous broadleaved forest community. This transition reflects a fundamental ecological question concerning temporal changes in species composition, making PKF suitable for applying the SFTS approach to investigate long‐term successional processes (Tang et al. 2013; Ashton and Zhu 2020). Meanwhile, secondary pine forests such as PKF are increasingly becoming major components of global forest biomes and are facing challenges of adaptation and recovery under climate change (Zhou et al. 2024; Tudor et al. 2025). In this context, the PKF, which is distributed widely across the subtropical regions of southwestern China, provides a valuable model system for studying pine forest succession, with both regional specificity and global significance. Studies have been conducted on PKF's phylogeny (Zhao et al. 2018), aboveground biomass (Li et al. 2018; Fan et al. 2024), and carbon storage (Gao et al. 2021); however, few studies have explored the community assembly patterns throughout its successional process. To achieve a better understanding of the mechanisms underlying pine forest community assembly under climate change, we analyzed the changes in TD and PD during PKF succession on the basis of the SFTS approach. We aim to understand: (1) How similar are TD and PD at each successional phase, and how do potential driving factors shape them? (2) Do successional shifts in TD and PD show dissimilar community assembly processes? (3) What role does the spatial effect inherent in the SFTS approach play in explaining temporal variation? Although this study is geographically restricted to Yunnan, PKF shares similar ecological and evolutionary backgrounds with the subtropical pine forests widely distributed from Southeast Asia to South Asia. Therefore, the findings of this study not only enhance our understanding of the community assembly mechanisms of pine forests during succession, but also provide general insights into the successional dynamics of subtropical pine forests worldwide under the influence of climate change and human activities.

## 2 Materials and Methods

### 2.1 Study Area

**FIGURE 2:**
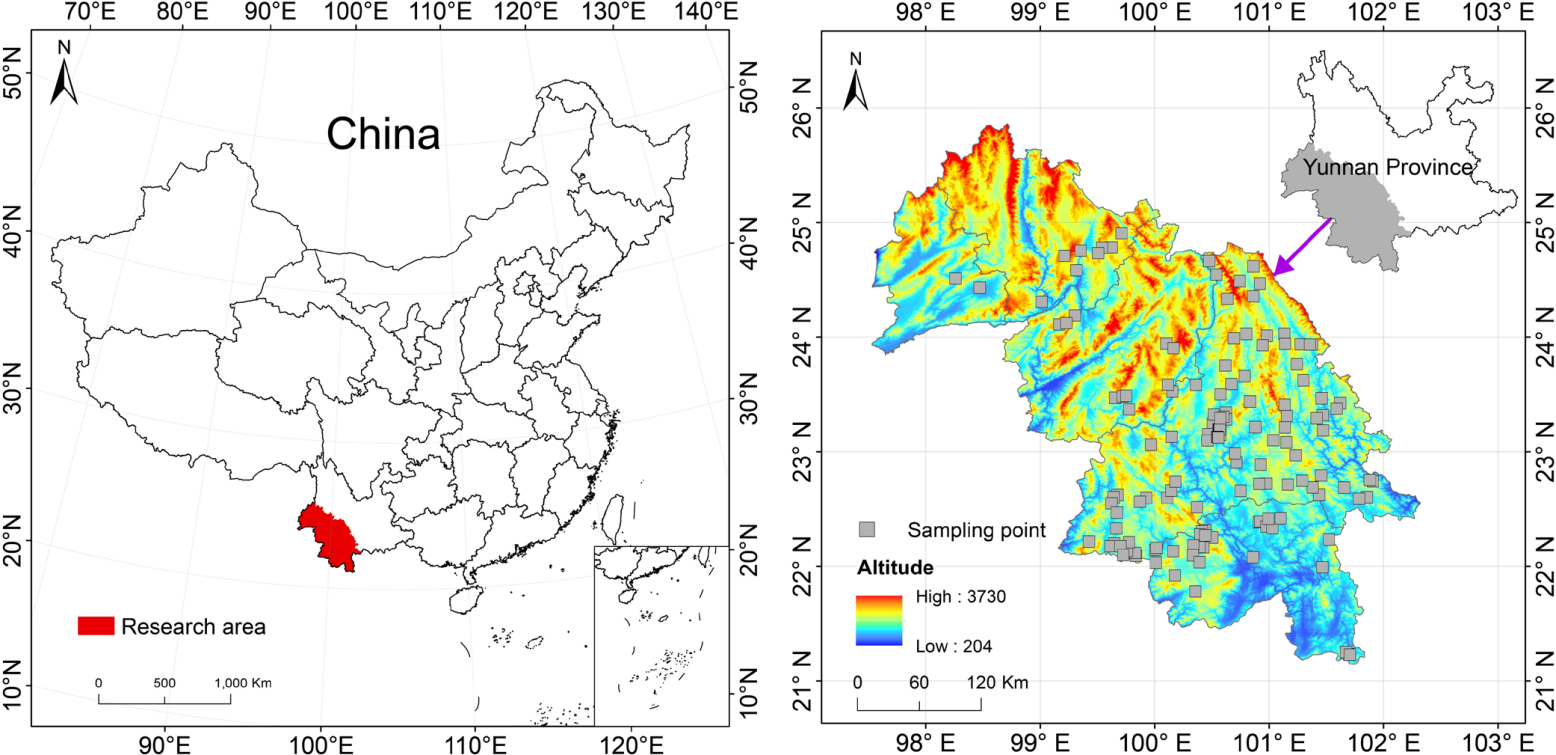
Research area and sampling plots of PKF.

This study was conducted in southwestern Yunnan Province, China (Figure 2), covering the geographic range from 21°05′–25°47′ N and 98°08′–102°25′ E. The region experiences a subtropical monsoon climate with distinct dry and wet seasons. The terrain slopes from north to south, resulting in notable topographic heterogeneity. Climatically, the area exhibits a large diurnal temperature range (mean annual temperature: 17.6°C) coupled with a narrow annual temperature variation. Annual precipitation averages 1270 mm, concentrated primarily during the wet season (Yang 1990). Precipitation exhibits pronounced seasonality, with approximately 85% of the annual amount occurring between May and October. The region has an accumulated annual temperature of 6400°C–7300°C and receives 2281–2453 h of sunshine per year. Soils are classified as acidic red soils, typical of subtropical monsoon climates.

### 2.2 Sampling Design

From 2021 to 2023, we conducted a comprehensive survey of PKF in Yunnan using a stratified–spatially constrained sampling design on the basis of the typical plot method (Wang, Fang, et al. 2020). We first identified the continuous distribution area of PKF using Global Biodiversity Information Facility (GBIF, https://www.gbif.org/) and Chinese Virtual Herbarium (CVH, https://www.cvh.ac.cn/) databases, combined with forest resource inventory data and remote sensing imageries, and used the forest distribution as the primary stratum. On this basis, elevation, slope, aspect, and landform type were further considered to ensure that the plots covered the major environmental gradients. To minimize the influence of spatial autocorrelation, all sampling plots were separated by a minimum distance of at least 5 km. The size of each plot was defined as 20 m × 30 m. Each woody plant with a diameter at breast height (DBH) ≥ 2.5 cm and a height greater than 1.3 m was measured. The species name, individual count, base diameter, DBH, height, crown size, and growth status of the trees were recorded. Furthermore, we calculated tree density (ind./ha), tree cover (%), and stand height (m), which provide essential information for understanding community changes during forest succession (Table S1). As for shrub and herb species, five 5 m × 5 m and 1 m × 1 m subplots were established in the four corners and center of the main plot. Within these subplots, all plant species were identified, and for each species, base diameter, crown size, height, coverage, and individual number were measured. Additionally, any shrub or herb species present within the main plot but not encountered in the subplots were also recorded. A total of 144 plots were investigated in this study (Figure 2).

### 2.3 Classification of Successional Stages and Inference of Stand Age

Simao pine, as a typical pioneer tree species, often exhibits clustered and synchronous regeneration (Liu et al. 2024). Therefore, the age of the largest individual within a stand is generally considered a reliable proxy for the overall stand age (Alberdi et al. 2013). In this study, we employed the SFTS approach by selecting stands representing different successional stages to construct a successional sequence (Fu et al. 2023). The age range of each PKF stand was inferred on the basis of putative DBH–age relationships, supplemented by local forestry records, consulted with forestry or nature reserve managers, and interviewed with nearby villagers (Li 2010; Tang et al. 2020). Without doubt, this method involves a certain degree of uncertainty, mainly arising from microhabitat‐driven variation in individual growth and recall bias in interview‐based information. To assess and minimize these uncertainties, we implemented the following measures before conducting data analyses: (1) a plot was included only when the interview information was cross‐validated with the DBH structure of trees in the stand; and (2) considering the potential methodological errors, we set an age estimation uncertainty of ±3 years. Then five stage groups were classified according to the Age‐class and Age‐group Division Standards for Dominant Tree Species (LY/T 2908–2017, https://std.samr.gov.cn/), an official forestry industry standard promulgated by National Forestry and Grassland Administration of the People's Republic of China, that is, young forest (≤ 20 years old, *N* = 12), middle‐aged forest (21–30 years old, *N* = 27), near‐mature forest (31–40 years old, *N* = 37), mature forest (41–60 years old, *N* = 41), and overmature forest (≥ 61 years old, *N* = 27). *N* is the number of plots surveyed in each group. It is important to note that although the young forest stage was defined according to the standard criteria of LY/T 2908–2017 stands, no sapling‐dominated plots or early post‐establishment cohorts were encountered during the field survey. All individuals in this category had DBH values typically ≥ 10 cm, indicating that these stands had already passed the highly dynamic initial regeneration phase. Therefore, the earliest stage in this study represents an early developmental phase after the sapling stage rather than newly regenerated stands. The plot information is shown in Table S1. This stage classification ensures overall inference regarding community assembly patterns during succession. It has been widely demonstrated to be reliable and effective in similar studies of secondary forest succession (Abbas et al. 2019). Additionally, the pine forest plots of different age classes can be used as spatial substitutes for a “time series” under the premise that PKF is predominantly distributed across the study area and the climatic background remains consistent. The plots are applicable to quantify the law of niche distribution at different stages, which is suitable as a substitute index for the SFTS method in the processes of succession, thereby revealing the laws of community succession (Oliver and Larson 1996; Zhang et al. 2021; da Costa et al. 2025; Lanta et al. 2025).

### 2.4 Explanatory Variables

The bioclimate data were derived from the WorldClim database (http://www.worldclim.com/, accessed on 9 March 2024), including 19 bioclimatic factors. Topographic data (elevation, aspect, slope) and geographic coordinates were collected using handheld GPS receivers for all plots. Plot‐specific fire occurrences were recorded during field surveys and verified using local forest management archives. Monthly burned area data were sourced from the MODIS database (MCD64A1.061, https://developers.google.cn/earth‐engine/datasets/catalog/MODIS_061_MCD64A1, accessed on 9 March 2024). Burned area data were aggregated to annual averages using the GEE cloud computing platform (https://earthengine.google.com/, accessed on 10 March 2024) for subsequent analysis. During field surveys, we recorded plot‐level signatures of human disturbance. Annual Mean Human Activity Index data for 2000–2020 were retrieved from the Data Center of the Chinese Academy of Sciences (https://figshare.com/, accessed on 9 March 2024). Because the field‐based wildfire and human footprint data were collected as discrete ordinal variables, their applicability in quantitative analyses requiring continuous predictors was limited. To reduce the subjectivity and observer's bias inherent in discrete, plot‐level disturbance scoring and to retain more quantitative information, we additionally extracted continuous wildfire and human footprint layers from spatial databases and subsequently evaluated the correlation between the field‐measured disturbance levels and the corresponding database values. The results showed a significant and consistent correlation between the two datasets (Figure S1), indicating that the database variables reliably capture the disturbance gradients observed in the field. Therefore, we used the remote‐sensing‐based data of wildfire and human footprint. The spatial resolution of the above environmental data is 1 × 1 km. Using the GPS‐recorded latitude and longitude of each field plot, environmental data were extracted in ArcGIS (version 10.7). The explanatory variables were chosen as reliable proxies for key regional‐scale environmental gradients, also applicable at the plot scale. A critical feature of our sampling strategy is that the minimum distance between any two plots exceeds 5 km, ensuring that each plot is spatially independent from the 1 km grid environmental data. The plot network was effectively transformed into a discretized sampling of broad‐scale environmental gradients. Macroscale climatic variables represent long‐term environmental constraints that determine species' physiology, colonization, and successional trajectories, even at fine spatial scales. Thus, these variables do not represent microhabitat conditions; rather, they capture the distinct regional context of each plot, including climatic limitations, disturbance history, and the impacts of anthropogenic activities. These contextual factors filter the potential species pool and systematically influence the successional pathways observable at each plot. Such use of environmental variables to investigate regional climatic drivers of successional processes has been widely adopted (Janečková et al. 2024).

Because ecological and environmental data are derived from the Earth's surface, spatial autocorrelation among sampling points is often observed (Borcard et al. 2011). Spatial variables are represented by eigenvectors derived from the principal coordinates of the neighborhood matrix (PCNM) on the basis of community geographic coordinates (Borcard and Legendre 2002; Borcard et al. 2004). PCNM allows the spatial impact on local community development to be evaluated on multiple scales. In order to study the explanatory account of spatial effects, the spatial information data in our study were first transformed into the Cartesian coordinate system on the basis of ArcGIS software. We constructed a full model using redundancy analysis (RDA) and performed forward selection to obtain 16 principal component axes with significant positive eigenvalues for further variable screening (Table S2). A geographic distance matrix was also calculated using Euclidean transformation to serve as the explanatory variables of spatial characteristics. Spatial variables were generated using the “dbmem” function in the “adespatial” package (Guénard and Legendre 2022).

To avoid multicollinearity among explanatory variables, we first assessed pairwise relationships using Spearman's correlation (Figure S2). Subsequently, on the basis of canonical correspondence analysis (CCA) contribution rates and interpretability, we removed variables with low contributions and high intercorrelations. We applied a Hellinger transformation to the species‐abundance data. Then, we computed variance inflation factors (VIFs) and iteratively removed the variable with the highest VIF if it exceeded 5, repeating this process until all remaining VIFs were ≤ 5. The significance of both the overall model and individual environmental factors was assessed (Luo et al. 2023). Finally, 22 explanatory variables meeting the criteria (absolute Spearman's correlation coefficient < 0.8 and VIF ≤ 5) were retained (Bolded in Table S3).

### 2.5 Selection and Calculation of Diversity Metrics

To comprehensively characterize changes in community features and assembly mechanisms during PKF succession from multiple dimensions, we employed a multidimensional set of diversity metrics, including taxonomic, phylogenetic, and structural indices. These metrics capture different aspects of community structure and complement one another in revealing underlying patterns. The indices are introduced in detail in the following subsections.

#### 2.5.1 Taxonomic Diversity Metrics

Species richness, Shannon–Wiener index, Simpson index, and Pielou evenness index were used to evaluate the species diversity of PKF at different successional stages. The above metrics describe the current species composition and abundance distribution of the community, reflecting species richness, patterns of individual abundance, and dominance structure. They are highly sensitive to species turnover and changes in abundance patterns during succession, and therefore provide complementary information on community structure that cannot be captured by phylogenetic metrics alone (Lanta et al. 2023). The calculation formulas for these diversity indices are as follows: (Simpson 1949; Spellerberg and Fedor 2003; Zhang et al. 2012):

(1) Species richness index:R=S

(2) $$\text{Shannon−Wiener index}:H=\;–\sum_{i=1}^{s}\left(P_{i}\ln P_{i}\right)$$

(3) $$\text{Simpson index}:D=1{–\sum }_{i=1}^{s}{\left(N_{i}/N\right)}^{2}$$

(4) $$\text{Pielou evenness index}:J=H/\ln\left(S\right)$$

where *S* is the total number of species in the community; *P*_*i*_ is the proportion of the total abundance of the *i*th species; *N*_*i*_ is the sum of the number of a certain species *i* in the plots; *N* is the total number of individuals of this species in the community plots.

The Sørensen dissimilarity index was used to quantify taxonomic β diversity (TβD) of plant communities in the study area, because it is widely used in vegetation ecology and provides a robust measure of species turnover, particularly suitable for examining community replacement processes driven by succession (Baselga 2010; Duan et al. 2024). Because TβD does not incorporate phylogenetic relationships, it provides a critical reference against which phylogenetic β diversity (PβD) can be compared. This allows us to distinguish whether community differences arise primarily from species turnover itself or from phylogenetic divergence associated with successional differentiation. The equation is calculated as follows:

(5) $$\text{Taxonomic Sørensen index}\;\left(\mathrm{T\beta D}\right)=\left(B+C\right)/\left(2A+B+C\right)$$

where *A* is the number of individuals of common species in community B and community C; *B* and *C* are the number of species individuals unique to community B and community C, respectively.

#### 2.5.2 Community Stability Index

The average variation degree (AVD) was used to evaluate the community stability of PKF, and it is a stability metric that allows comparisons across different successional stages (Xun et al. 2021). It has been demonstrated in multiple studies to effectively capture fluctuations in community abundance, and it reflects community organization from both functional and structural perspectives (Xun et al. 2021). The AVD values were calculated by the following equation:

(6) $$\mathrm{AVD}=\left(\sum_{i=1}^{n}|x_{i}–{\bar{x}}_{i}|/\delta_{i}\right)/\left(k\times n\;\right)$$

Here, $x_{i}$ denotes the rarefied abundance of the species in a single sample, ${\bar{x}}_{i}$ is the mean value of $x_{i}$, and $\delta_{i}$ signifies the standard deviation of rarefied abundances within each sample group. k denotes the number of samples within a sample group, and n represents the number of species in each sample group. A smaller AVD indicates higher community stability. In this study, we used 1 − AVD to represent community stability, such that higher values correspond to more stable communities.

#### 2.5.3 Construction of Phylogenetic Tree

The “Plantlist” package was used to batch‐query the family and species information for all species recorded in the plots (Zhang et al. 2019). A phylogenetic tree was constructed for subsequent analysis using the “V.PhyloMaker” package (Jin and Qian 2019). Figure S3 shows the phylogenetic tree of the communities across the five successional stages.

#### 2.5.4 Phylogenetic Diversity and Phylogenetic Structure

PD represents the sum of branch lengths in a phylogenetic tree connecting all species within a community, reflecting their shared evolutionary history. It serves as a fundamental metric for quantifying phylogenetic diversity (Faith 1992). The nearest taxon index (NTI) was used to infer community assembly processes (Webb et al. 2002). NTI quantifies phylogenetic community structure by measuring the clustering of closely related taxa at the tips of the phylogeny (Swenson et al. 2006). In this study, assuming phylogenetic conservatism of functional traits (Wiens et al. 2010; Li et al. 2022), we interpreted NTI values as follows: NTI > 0, phylogenetic clustering (closely related species co‐occur), suggesting environmental filtering dominance. NTI < 0, phylogenetic overdispersion (distantly related species coexist), suggesting competitive exclusion dominance. If NTI = 0, no significant deviation from random assembly, indicating stochastic processes (Webb et al. 2002). PD and NTI are primarily used to describe the evolutionary dimension of communities. The calculation was implemented in the “pd” function and the “ses.mntd” function of the “picante” package (Kembel et al. 2010). The calculation formula is as follows:

(7) $$\mathrm{PD}=\sum_{\left(c\in C\right)}L_{c}$$

(8) $$\mathrm{SES}.\mathrm{PD}={\mathrm{PD}}_{\text{observed}}-\text{mean}\;\left({\mathrm{PD}}_{\text{random}}\right)/{\text{sdPD}}_{\text{random}}$$

(9) $$\mathrm{NTI}=-1\times \left({\text{MNTD}}_{s}-{\text{MNTD}}_{\mathrm{mds}}\right)/\mathrm{SD}\;\left({\text{MNTD}}_{\mathrm{mds}}\right)$$

where *C* is the minimal phylogenetic subtree connecting all species in the community, c represents a branch in C, and *L*_*c*_ is the branch length of c. SES.PD calculates the standardized effect size of PD. PD_observed_ represents the observed PD in a community, PD_random_ is the mean PD from null communities, and sdPD_random_ is the standard deviation of PD in null communities. We utilized the “taxa.labels” null model and performed 999 iterations to satisfy statistical criteria (Jarzyna et al. 2021). MNTD_s_ represent the mean phylogenetic distance between each species and its closest relative in the observed community. When 999 null model simulations were used to generate random communities, MNTD_mds_ is the mean MNTD from null communities. SD (MNTD_mds_) stands for the standard deviation of MNTD in null communities (Webb et al. 2002).

The phylogenetic Sørensen index was used to quantify phylogenetic dissimilarity between communities (Stegen et al. 2012; Duan et al. 2024). It focuses on the sharing degree of phylogenetic branches among communities, representing PβD. The calculation formula is as follows:

(10) $$\text{phylogenetic Sørensen index}\;\left(\mathrm{P\beta D}\right)=1-\left(2L_{c}/L_{A}+L_{B}\right)$$

where *L*_*A*_ and *L*_*B*_ are the total branch lengths of the two communities, and *L*_*C*_ is the shared branch length.

Therefore, the combined use of taxonomic, phylogenetic, and community structural indices provides a multidimensional and complementary framework for understanding the community assembly mechanisms during PKF succession.

### 2.6 Data Analysis

The α and β diversity indices and AVD across five successional stages were compared using one‐way ANOVA, followed by Duncan's multiple‐range test to identify significant differences, with the significance level controlled at *p* = 0.05 for all multiple comparisons. To explore the relationship between α diversity and environmental factors, a correlation heatmap was generated on the basis of the Spearman correlation test using the “psych” package. We employed Spearman's correlation because of its robustness to non‐normal data distributions. Further, the detrended correspondence analysis (DCA) was used to judge the adoption mode of ordination analysis. When the gradient length of the first DCA axis exceeds 4.0, CCA is recommended; when it is below 3.0, RDA is more appropriate (Turktas et al. 2012). For intermediate gradient lengths (3.0–4.0), either CCA or RDA may be used (Bastow Wilson 2012). Finally, permutation tests (999 permutations) were performed on the first two ordination axes to assess their relationships with explanatory variables, on the basis of the results of DCA and CCA using the “decorana” and “anova.cca” functions in the “vegan” package. This data‐driven approach (DCA followed by CCA/RDA) ensures that the most appropriate unimodal or linear model is selected to reveal the relationships between community composition and environmental gradients.

To explore β diversity patterns during succession and their relationships with environmental factors, we first transformed β diversity indices into a distance matrix and constructed non‐metric multidimensional scaling (NMDS) ordination plots for both TβD and PβD. NMDS was selected for its ability to visualize complex community dissimilarities on the basis of any distance measure without assuming linear relationships. Statistical significance was assessed through 999 permutations of the Monte Carlo simulation. Furthermore, we used permutation tests to identify the key environmental factors that significantly influence β diversity, on the basis of the “envfit” function from the “vegan” package (Oksanen et al. 2019). Finally, Mantel tests were performed using the “mantel_test” function from the “linkET” package to explore the influence of environmental influences on β diversity at each successional stage (Tan et al. 2013). The Mantel test is appropriate here as it tests the correlation between two distance matrices, aligning with the β diversity data structure.

To investigate the influences of niche and neutral processes on β diversity patterns, we integrated bioclimatic factors, topographic factors, wildfire, and human footprint as environmental variables, and performed variation partitioning analysis (VPA) with spatial variables to quantify their relative contributions. VPA was specifically chosen to disentangle the pure and shared effects of environmental filtering (niche processes) and spatial dispersal (neutral processes) on community assembly. VPA was implemented using the “rdacca.hp” function from the “rdacca.hp” package (Lai et al. 2022). Additionally, we used linear regression to model how the explanatory power of environmental and spatial variables on β diversity changed throughout succession. Finally, standardized taxonomic and phylogenetic diversity indices were employed to examine variations in α and β diversity across successional stages, whereas polynomial regression was applied to evaluate relationships between diversity metrics and community stability. Polynomial regression was used to allow for the detection of potential non‐linear (e.g., hump‐shaped or U‐shaped) relationships between diversity and stability, which are theoretically expected during succession. In this study, box plots and polynomial fitting curves were generated using Origin 2024 software, and all other analyses were conducted in R version 4.4.1 (https://www.r‐project.org; R Core Team 2024).

## 3 Results

### 3.1 Changes in Community Structure and Taxonomic and Phylogenetic α Diversity of PKF During Succession

Community structure properties showed clear and significant differences among the five successional stages (Figure S4). Tree density exhibited a general decreasing trend from young to overmature forest (Figure S4a), with young forest having significantly higher tree densities compared with mature and overmature forest. Tree coverage also declined with succession (Figure S4b), with young and middle‐aged forest showing significantly higher canopy cover than later successional stages. In contrast, stand height increased markedly along the successional gradient (Figure S4c).

**FIGURE 3:**
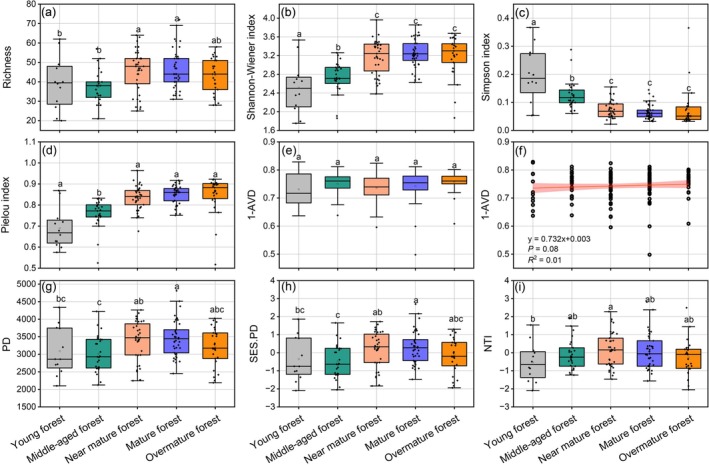
α diversity, community stability index, and phylogenetic structure of PKF at different successional stages. AVD, average variation degree; NTI, nearest taxon index; PD, phylogenetic diversity.

Taxonomic α diversity (TαD) of PKF significantly changed during succession. The species richness (SR) in near‐mature forest and mature forest was significantly higher than in young forest and middle‐aged forest (*p* < 0.05, Figure 3a), whereas the SR in overmature forest declined non‐significantly (*p* > 0.05, Figure 3a). The Shannon–Wiener index of young forest and middle‐aged forest was significantly lower than that of other stages (*p* < 0.05, Figure 3b). Throughout succession, the Simpson index decreased significantly, with young and middle‐aged forest showing higher values (*p* < 0.05, Figure 3c). The Pielou evenness index increased significantly and was lower in young and middle‐aged forest (*p* < 0.05, Figure 3d). Although community stability showed no significant differences among stages, it increased progressively during succession (Figure 3e,f).

Similar to SR patterns, both PD and SES.PD increased during the early succession and then declined. PD and SES.PD in the young forest and the middle‐aged forest were significantly lower than those in the mature forest (*p* < 0.05, Figure 3g,h). During succession, the NTI first increased and then decreased (Figure 3i). NTI values in young forest and middle‐aged forest were less than 0, in near‐mature forest were greater than 0, in mature forest were near 0, and in overmature forest were less than 0. This trajectory reflected a shift from phylogenetic overdispersion to clustering, then to randomness, and back to overdispersion. NTI dynamics demonstrated that different community assembly mechanisms dominated the local communities at different successional stages (*p* < 0.05, Figure 3i).

### 3.2 Taxonomic and Phylogenetic β Diversity of PKF During Succession

**FIGURE 4:**
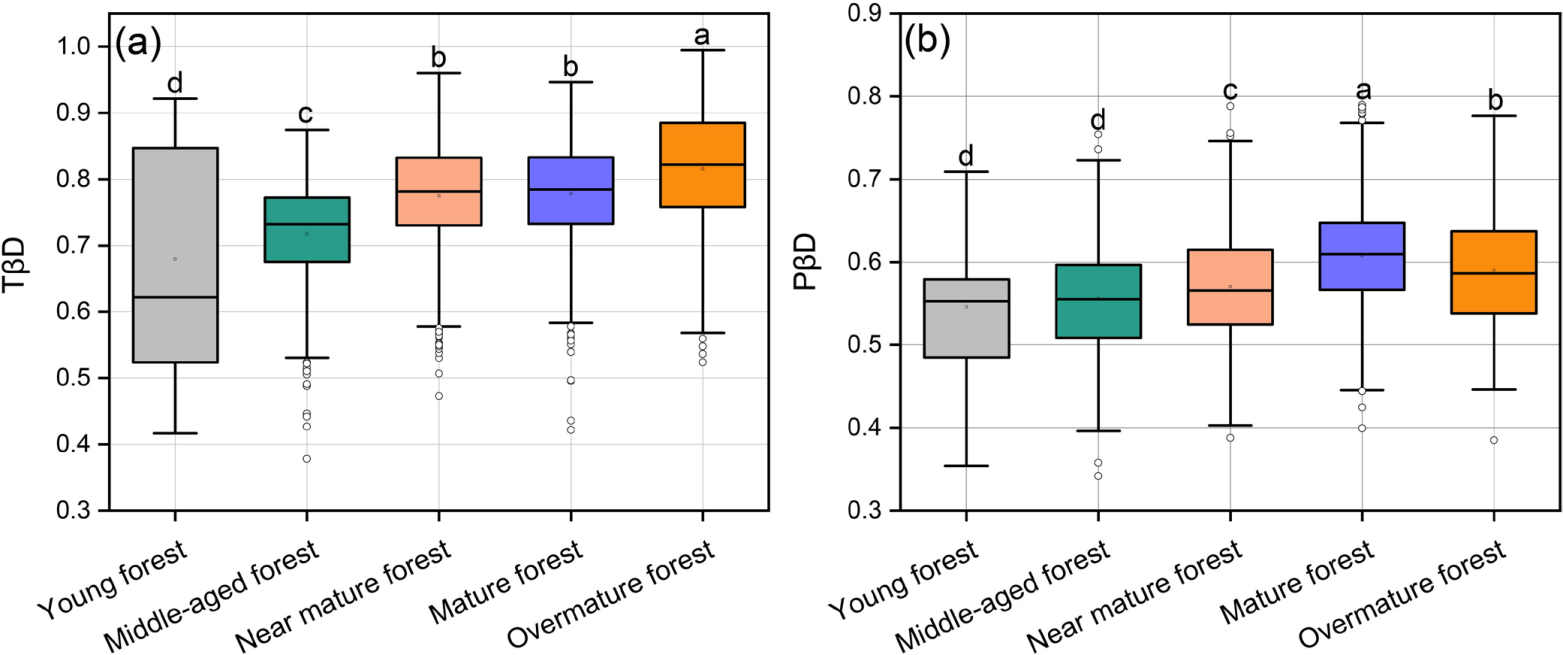
Taxonomic β diversity (TβD) and phylogenetic β diversity (PβD) of PKF at different successional stages.

According to the Sørensen dissimilarity index of PKF at different successional stages (Figure 4a), TβD exhibited a monotonic increasing trend. In Figure 4b, PβD showed a fluctuating pattern, first increased and then decreased. PβD reached the maximum in the mature forest, which was significantly higher than at all other stages (*p* < 0.05). Moreover, PβD remained lower than TβD throughout all successional stages (Figure 4).

### 3.3 Relationship Between α Diversity and Explanatory Variables

**FIGURE 5:**
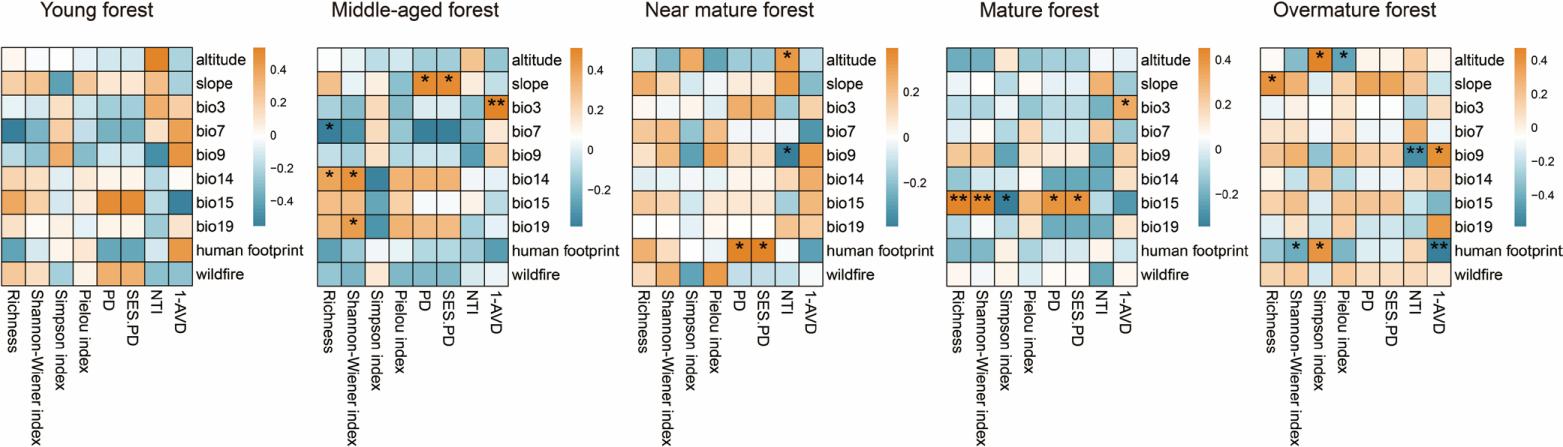
Correlation between diversity index and explanatory variables. Significance levels: **p < 0.01, *p < 0.05.

At different successional stages, the correlations between α diversity and explanatory variables were distinct (Figure 5). In the young forest, no α diversity or stability indices correlated significantly with environmental variables. In the middle‐aged forest, SR was negatively correlated with bio7 but positively correlated with bio14. The Shannon–Wiener index was positively correlated with bio14 and bio19. PD and SES.PD were positively correlated with slope. The stability index was positively correlated with bio3. In the near‐mature forest, PD and SES.PD were positively correlated with human footprint. NTI was positively correlated with altitude but negatively correlated with bio9. In the mature forest, SR and the Shannon–Wiener index were positively correlated with bio15, whereas the Simpson index was negatively correlated with bio15. PD and SES.PD were also positively correlated with bio15, and the stability index was positively correlated with bio3. In the overmature forest, SR was positively correlated with slope. The Shannon–Wiener index was positively correlated with human footprint, whereas the Simpson index was negatively correlated with both the human footprint and altitude, and the Pielou evenness index was negatively correlated with altitude. NTI was negatively correlated with bio9. The stability index was positively correlated with bio9, but negatively correlated with the human footprint. In general, the effects of environmental factors on forest diversity depended on the successional stage. Human impacts mainly occurred at later stages, and wildfire had no significant effect on TD and PD change during succession.

**FIGURE 6:**
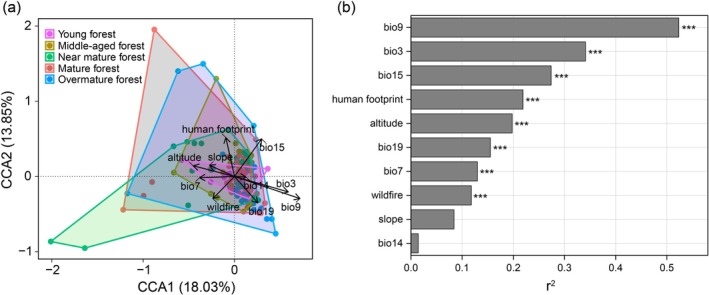
CCA ordination results and permutation test of PKF.

We first conducted DCA ordination and found that the first‐axis gradient length exceeded 4.0 (Table S4). Therefore, CCA ordination was used to explore the dominant drivers of local‐scale successional processes. The successional process exhibited a heterogeneous pattern that changed progressively under specific environmental drivers (Figure 6a). Permutation test results indicated that bio9 was the key environmental factor driving the successional dynamics of PKF (Figure 6b).

### 3.4 Relationship Between β Diversity and Explanatory Variables

**FIGURE 7:**
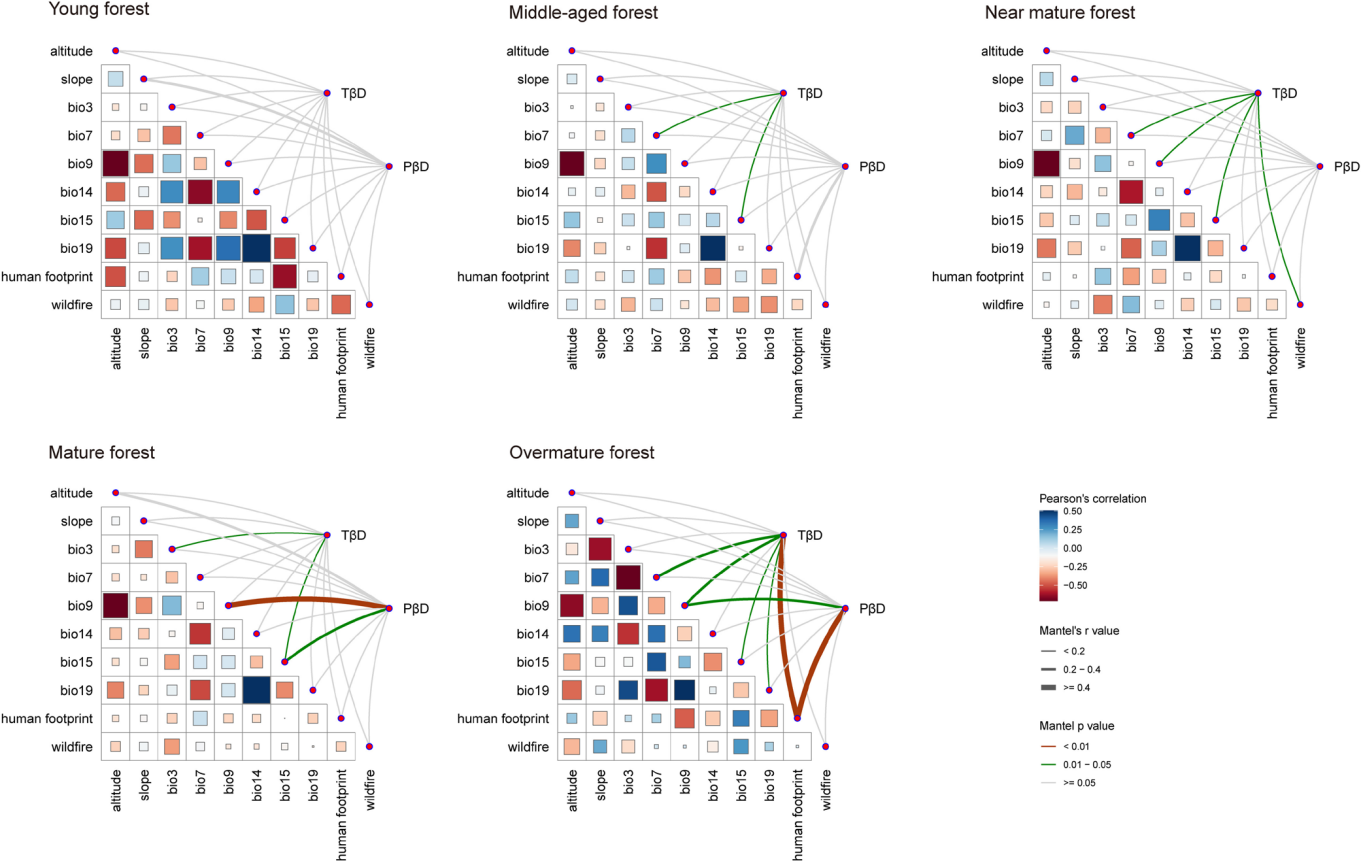
Mantel test of taxonomic β diversity (TβD) and phylogenetic β diversity (PβD) at different successional stages.

On the basis of the Mantel test, we analyzed the dominant environmental factors influencing TβD and PβD at each successional stage (Figure 7; Table S5). The dominant environmental factors influencing β diversity varied across successional stages from young to overmature forest. In the young forest, β diversity was not significantly correlated with any environmental factor. In the middle‐aged forest, TβD was significantly correlated with bio7 and bio15, whereas PβD showed no significant correlations. In the near‐mature forest, TβD was significantly correlated with bio7, bio9, bio15, and wildfire. In the mature forest, TβD was significantly correlated with bio3 and bio15, whereas PβD was significantly correlated with bio9 and bio15. In the overmature forest, TβD was significantly correlated with bio7, bio9, and human footprint, and PβD was significantly correlated with bio9 and human footprint. The influence of environmental factors on TβD and PβD was variable among successional stages and shifted with forest maturity. At early stages, β diversity patterns were primarily shaped by stochastic processes, whereas at later stages, human disturbance became the dominant driver.

**FIGURE 8:**
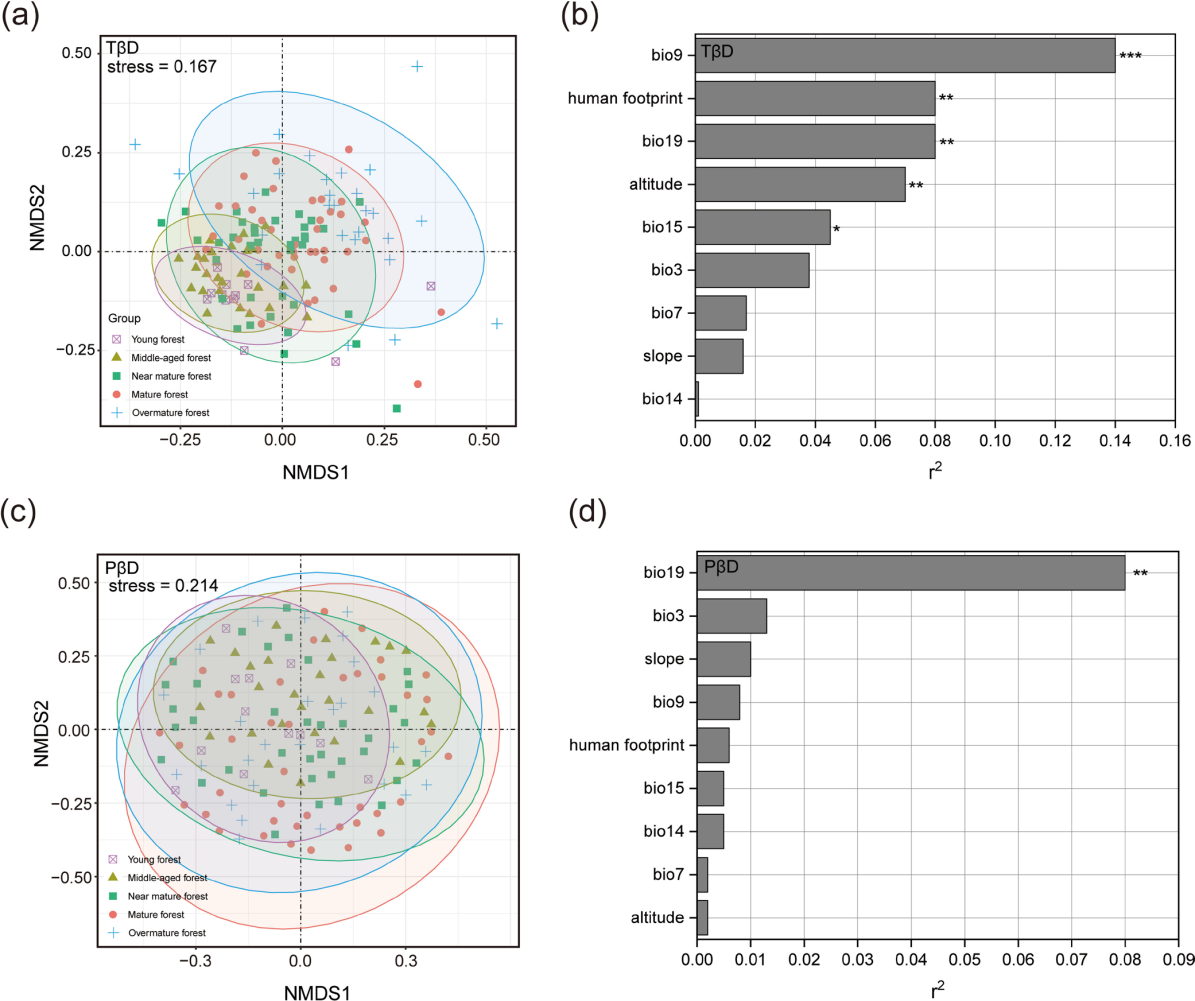
NMDS ordination diagram and permutation test of taxonomic β diversity (TβD) and phylogenetic β diversity (PβD) in PKF's succession processes.

NMDS and permutation tests were used to analyze the dominant driving factors of β diversity during succession at the regional scale. For TβD, the different successional stages were divided into five distinct clusters and gradually shifted from the third quadrant to the first quadrant (Figure 8a). The permutation test results indicated that bio9 was the main environmental driver of TβD during succession (Figure 8b). As the succession progressed, the degree of phylogenetic differentiation among PKF slightly increased (Figure 8c), and bio19 emerged as the primary driver of PβD (Figure 8d). Other environmental factors showed no significant contribution to PβD.

### 3.5 Effects of Spatial Factors and Environmental Factors on the Succession of PKF

**FIGURE 9:**
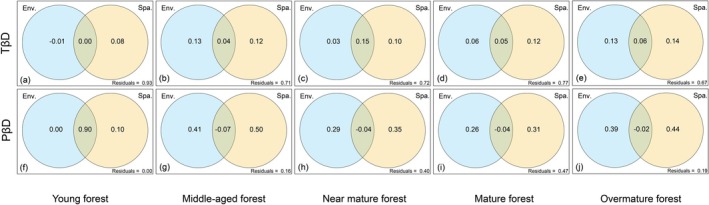
Variation partitioning of PKF community TβD and PβD on the basis of environmental factors and spatial factors. Env.—the partition in which environmental effects are explained separately, light blue; Spa.—the partition in which spatial effects are explained separately, light yellow; Interactive part refers to the partition in which environmental and spatial effects are explained together. PβD, phylogenetic β diversity; TβD, taxonomic β diversity.

VPA results showed that, except for the middle‐aged forest, the contribution of spatial factors to both TβD and PβD was greater than that of environmental factors at all other successional stages (Figure 9). Furthermore, by fitting the explanatory power of spatial factors and environmental factors at each successional stage, we found that spatial factors consistently explained more variation than environmental factors (Figure S5). However, the explanatory power of spatial factors gradually declined as succession progressed, whereas that of environmental factors increased significantly (Figure S5).

## 4 Discussion

### 4.1 Changes of α and β Diversity in Succession Processes

**FIGURE 10:**
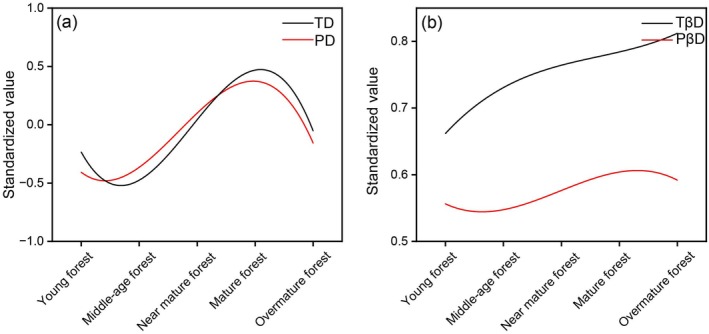
Standardized α and β diversity in taxonomy and phylogeny of PKF during succession. PD, phylogenetic diversity; PβD, phylogenetic β diversity; TD, taxonomic diversity; TβD, taxonomic β diversity.

The TD and PD of PKF both followed a cosine‐shaped pattern (Figures 3a,g and 10), a trajectory that transcends the traditional framework of linear or hump‐shaped curves and reveals diversity fluctuations driven by multiple stages and mechanisms, which can be implied as an extension of the classic “hump‐shaped diversity curve” (Geng et al. 2022). These results align with species diversity trends observed during the succession of abandoned lands in southern Illinois, USA (Bazzaz 1968; Kassen et al. 2000). At the early stage of succession, although niches were not saturated, the extremely high tree density likely intensified intraspecific competition for limited resources (Figure S4a), resulting in unstable community habitats, low niche differentiation, and less survival of pioneer species. This may explain the lower levels of both TD and PD (Chai et al. 2016; Fu et al. 2023). The priority effect led to a strong stochastic process (Figure 9; Viana et al. 2016), which explains the lack of significant relationships between species diversity and environmental factors (Figure 5). The succession progresses with decreasing tree density and increasing stand height (Figure S4a,c). The microclimatic and micro‐environmental conditions within communities gradually change, allowing more species to colonize. Intense niche differentiation promotes increases in both TD and PD, including the establishment of more shade‐tolerant species in the understory (Lebrija‐Trejos et al. 2016). In the later phases of succession, light becomes a more limiting factor. With the community structure shifting toward a taller and more open canopy (Figure S4b,c), light limitation is intensifying through deepening vertical shading, in consistent with poor understory light conditions during late succession (Baldissera et al. 2014). Meanwhile, niche saturation and limitations imposed by precipitation‐related factors intensify interspecific competition (Figure 7). There is the exclusion or elimination of some species by dominant ones (Selaya and Anten 2010), which in turn causes a decline in both TD and PD (Figure 3). During succession, gradual increases in species adaptation (Isbell et al. 2009) and niche differentiation (MacArthur and Levins 1967) lead to gradual increases in community evenness and stability (Figure 3e,f; Naeem and Li 1997).

**FIGURE 11:**
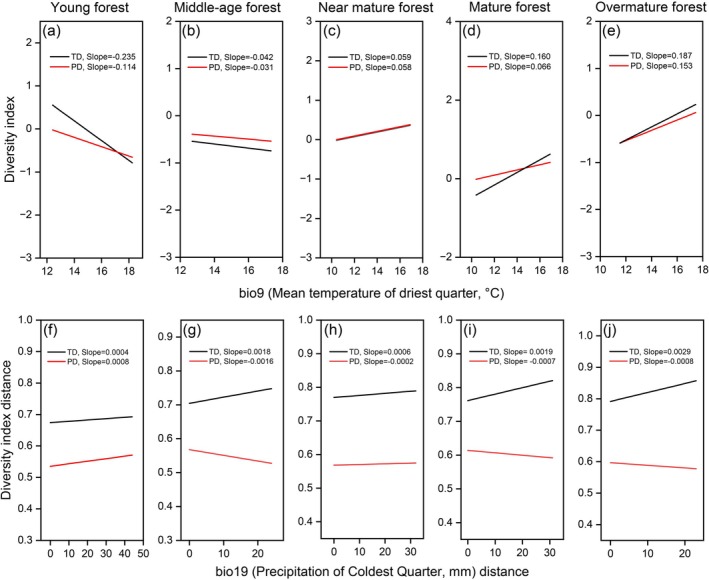
Response of taxonomic diversity (TD) and phylogenetic diversity (PD) to dominant environmental factors at different successional stages of PKF.

Further analysis revealed a synchronous time‐lag effect between TD and PD during succession (Figure 10a). Regarding β diversity, an asynchronous pattern was observed between TβD and PβD (Figure 10b), revealing a decoupled response of ecological and evolutionary processes to driving factors throughout succession. Successional changes directly affect TD (Figures 5 and 11). However, the existence of redundant species and the emergence of new lineages may strongly impact the phylogenetic structure of the region. These new lineages are the first to adapt and expand into new environments without immediately altering TD (Cavender‐Bares et al. 2006). With niche differentiation or the earlier filling of niche gaps (Cadotte et al. 2012), the available niches are rapidly occupied, leading to earlier changes in PD (Wang, Lv, et al. 2020, 2022). In contrast, TD reflects the number of coexisting species, which typically changes on an ecological timescale as it depends on the outcomes of ecological interactions and competition among species. This dependence usually results in TD changes lagging behind PD changes (Tilman 1994; HilleRisLambers et al. 2012). In terms of β diversity, TβD fluctuates more frequently, but many species replacements occur within closely related lineages, resulting in smaller changes in PβD (Graham and Fine 2008). Species turnover intensifies during the middle to late successional stages, driving the observed monotonic increase in TβD (Chase 2007). Generally, the regional species pool remains relatively stable, especially within the same plant community type (such as coniferous forests or grasslands). Even with species replacement, many of the newcomers still belong to closely related lineages, which constrains fluctuations in PβD (Cavender‐Bares et al. 2004). Therefore, the cosine fluctuation pattern of PβD reflects both the size and the evolutionary stability of the regional species pool in PKF (Figure 10b).

### 4.2 Inferences of Community Assembly in Succession Processes and Its Drivers

Given that phylogenetic clustering and overdispersion are respectively explained by environmental filtering and competitive exclusion (Emerson and Gillespie 2008), we infer that PKF's community assembly is primarily driven by competitive exclusion in the early stage, by environmental filtering in the mid‐successional stage, and again by competitive exclusion in the later stage (Figure 3i). This pattern extends the traditional framework of linear and unidirectional community assembly mechanisms in succession theory, revealing that environmental filtering and competitive exclusion are not simply alternative processes, but rather interact dynamically with changes in stand structure, resource availability, and evolutionary constraints. In the early successional stage, light transmittance under the forest is high, and the low species diversity may be due to the competition for light (Li, Peng, and Dang 2013). In the near‐mature forest, environmental conditions tend to be stable, and this stable environment strengthens the sorting for species with specific traits or lineages. On the other hand, both TD and PD reach their peak, suggesting the niche may be saturated. Therefore, environmental filtering plays a dominant role in shaping the phylogenetic structure (Figure 3i). In addition, the “intermediate disturbance” hypothesis may also play a role (Figure 5; Jiang et al. 2017). As succession progresses to the later stages, the community structure tends to stabilize (Figure 3f). High niche overlap leads to intense utilization of resources and increased competition pressure among species within communities (Webb et al. 2002; Kraft et al. 2008; Mayfield and Levine 2010; Fu et al. 2023). This pattern of community assembly during succession has been observed in the coniferous and broadleaved mixed forest of Changbai Mountain (Hou et al. 2017) and in tropical rain forests (Mo et al. 2013). However, on the Loess Plateau, environmental filtering is dominant in the later successional stages (Chai et al. 2016). This difference may be related to the aridity of the environment, specifically, a strong dependence on water availability.

Temperature‐related factors (bio9) directly affect variation in species composition during succession in PKF (Figures 6b and 8b). Temperature directly determines the colonization, growth, maturation, and survival of species (Sánchez‐González and López‐Mata 2005), greatly resulting in different species composition across regions or at different successional stages (Chen and Lewis 2024). *P. kesiya* var. *langbianensis* is a warm‐adapted coniferous tree species with a short evolutionary history (Gu and Li 1982); the mean annual temperature in its distribution area is 17.5°C–20.1°C, consistent with its biological characteristics (Li, Su, et al. 2013). In addition, the human footprint also plays a significant role in the successional processes (Figures 6b and 8b), which may be related to increased habitat fragmentation and the colonization of invasive alien species (Hoffmeister et al. 2005). PD reflects deeper evolutionary relationships shaped by paleoclimate. Sporopollenin records from the Ice Age indicate that global cooling was generally accompanied by decreased precipitation (Bartlein et al. 2011). However, because of the unique geographical location of Yunnan Province and its complex topography, the Quaternary Ice Age had less impact on the vegetation in this area (Bartlein et al. 2011; Tang et al. 2018). With the continuous uplift of the Qinghai‐Tibet Plateau in the Neogene and the gradual strengthening of the southern Asia monsoon climate, *P. prekesiya* began to differentiate into *Pinus yunnanensis*, which is gradually adapted to a more arid environment, and *P. kesiya* var. *langbianensis*, which persisted in more humid habitats (Xing et al. 2010). Precipitation‐dependent factors (bio19) continue to play a role in shaping the phylogenetic structure of PKF during contemporary succession (Figure 8d). This decoupling mechanistically explains how contemporary succession is simultaneously constrained by both immediate abiotic conditions and deep evolutionary history.

### 4.3 Explanations of Taxonomic Diversity and Phylogenetic Diversity on Community Stability

**FIGURE 12:**
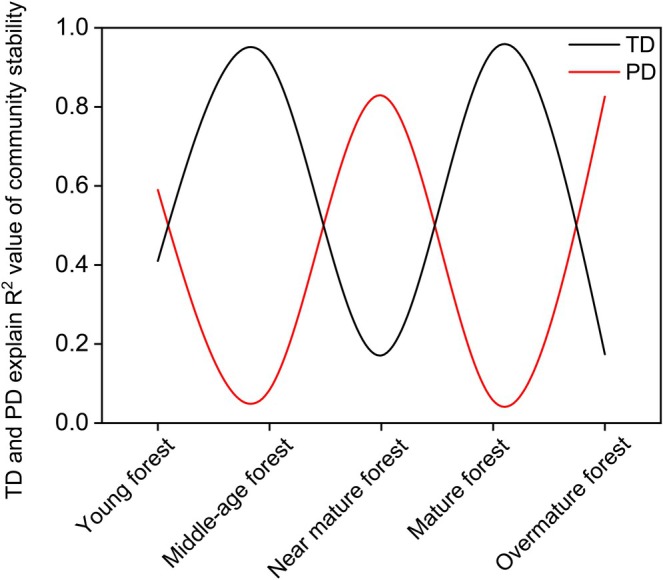
Relative explanatory ability of taxonomic diversity (TD) and phylogenetic diversity (PD) to community stability in PKF's succession.

Pine forests are inherently pioneer communities that will eventually be replaced by broadleaved forests (Anyomi et al. 2022). Yet, they have persisted for millions of years, owing largely to periodic disturbances that reset successional trajectories (Maleki et al. 2019). Traditionally, community stability has been explained by TD alone (MacArthur 1955; Bezemer and Van Der Putten 2007), but this view may underestimate ecosystem complexity (Santos et al. 2014; Liu, Ma, et al. 2021; Liu, Zhang, et al. 2021; Wei et al. 2024). During the succession process of the PKF communities, the explanatory power of TD and PD for community stability exhibits a dynamic pattern of alternating dominance (Figure 12). Redundancy in TD and PD within a community contributes to stability across different levels (Sadeghinia et al. 2023). TD enhances communities' short‐term functional stability through niche differentiation (Liu et al. 2023), whereas PD confers long‐term adaptability and stress resistance via evolutionary diversity (Sadeghinia et al. 2023). Their complementarity enables the community to maintain strong stability facing environmental changes at different time scales (Symstad et al. 2003). This multidimensional stability mechanism during succession may provide a novel perspective on the long‐term persistence of pioneer pine forests.

### 4.4 Relative Contribution by Spatial Variables and Environmental Variables

The significance of the spatial domain in ecological theory has been widely acknowledged (Legendre 1993; Borcard et al. 2004; Wu et al. 2021; Virgilio et al. 2022). Many ecologists mostly use the classic theory of SFTS in succession study (Parrish and Bazzaz 1982; Yuan et al. 2023). However, the spatial effects themselves are often overlooked. In this study, we divided the explanatory variables into two categories: environmental variables and spatial variables, and explored their effects on community structure from both species and phylogenetic perspectives during succession (He et al. 2022). Compared with spatial variables, environmental variables exert relatively less influence on species composition (Figure 9). However, as succession progresses, the influence of environmental variables gradually increases (Figure S5). In the early community, niches have not been fully occupied, random processes strongly influence community composition, and the environmental filtering effect is weak (Myers and Harms 2009). Species colonization is limited by the dispersal of pioneer species with close phylogenetic relationships (Weiher et al. 2011). The community stabilizes as succession progresses, and niches become saturated; then resource competitions are intensified. Species composition is increasingly determined by environmental adaptation, acting more strongly on specific evolutionary lineages (Cadotte and Tucker 2017). This pattern may reflect the “successional continuum hypothesis” (Gravel et al. 2006), which proposes that at the regional scale, both neutral processes and niche differentiation jointly shape community assembly, forming a continuum rather than a binary opposition. In addition, it is important to note that the unexplained variance at some succession stages in our study is high, underscoring the complexity of ecological processes (Legendre et al. 2009).

### 4.5 Limitation of the SFTS Method in Succession Studies

Although the SFTS method is widely used in many ecological studies, especially in studies of community succession (Blois et al. 2013; Kharouba and Williams 2024), this approach still has important limitations and should be applied with caution when interpreting results. Firstly, community spatial patterns may be shaped by both temporal and spatial processes (such as dispersal limitation and local interspecific interactions). Therefore, the SFTS approach may conflate temporal and spatial processes, making it difficult to determine whether observed community variation stems primarily from spatial structure or from temporal succession. To address this, our study introduces the “dbmem” function to disentangle temporal and spatial influences in the successional process (Figure 9). Secondly, SFTS often ignores the possible historical effects of each plot or community, such as the legacy effects of disturbance events (e.g., fire, human footprint) on the community structure. These historical events may not be correlated with temporal succession patterns along spatial gradients. Therefore, we explicitly incorporated two disturbance‐related variables into the analysis to minimize these potential sources of error. Our findings highlight that spatial variation should not be overlooked when applying the SFTS approach. However, the five successional stages formed a clear spatial continuum, and the patterns of community and environmental variation were highly consistent with theoretical expectations. Thereby, the applicability of the SFTS approach is supported in this context. Moreover, we carefully probed the inferred successional dynamics, acknowledging that our data represent a space‐for‐time comparison rather than a directly observed temporal sequence. While accounting for spatial connectivity and incorporating more detailed environmental variables and historical processes can help minimize biases, these measures may remain insufficient. The successional configuration of pine forests by SFTS will be helpful to conduct further validation and in‐depth analysis using long‐term time series data.

### 4.6 Suggestions on Management and Conservation of PKF

The resin secreted by *P. kesiya* var. *langbianensis* contains a variety of bioactive compounds such as monoterpenes, diterpenoids, flavonoids, and phenolic acids, which have antibacterial, anti‐inflammatory, antiviral, and antitumor activities (Liu et al. 2022). Moreover, as a fast‐growing tree species, it is highly valued for papermaking and timber production (Li, Peng, and Dang 2013). However, sustainable logging and resin production depend on the healthy development of PKF. Our results indicate that species richness is low and community stability is poor during early succession, and community assembly is dominated by competitive exclusion. So, forest management should focus on reducing competitive stress at early stages to promote community development. For example, community stability may be enhanced by selective thinning or removal of dominant individuals. At the middle stage, species diversity and stability reach higher levels, and environmental filtering plays a dominant role. Because this stage also coincides with the peak period for resin extraction, a regulated harvesting practice participated in by local stakeholders is necessary to maintain both high diversity and resource sustainability. The increasing diversity and recovery of PD at the late stage suggest that management should promote natural regeneration and, if necessary, artificially plant late‐successional species to maintain ecosystem resilience. Across all stages, establishing a long‐term monitoring system is crucial to link management practices directly to successional dynamics and to strengthen local community awareness of rational resin extraction and forestry resource protection. As a regionally important ecosystem, the protection of PKF is critical not only for biodiversity and ecosystem functioning but also for the local economy and community livelihoods. Overall, management practices integrated with successional patterns will ensure forest protection and sustainable usage.

## 5 Conclusion

This study integrates ecological and evolutionary perspectives and confirms the classic hump‐shaped pattern with diversity peaking in the mid‐successional stage. More importantly, we propose two novel patterns: a “synchronous time‐lag effect” between TD and PD and an “asynchronous oscillation” between TβD and PβD. The former reveals that ecological and evolutionary processes do not respond synchronously through time, whereas the latter shows that TβD increases monotonically during succession, whereas PβD exhibits a low‐amplitude cosine‐like fluctuation. These dual‐track patterns in subtropical pine forests provide new insights into the interplay between ecological filtering and evolutionary constraints. Next, we identify a periodic shift in the dominant community assembly mechanisms, that is, from early competitive exclusion to mid‐successional environmental filtering, and then back to competitive exclusion in the late stage. This oscillatory pattern expands the traditional view of succession as a linear transition and demonstrates that biotic and abiotic processes alternate repeatedly across temporal scales and jointly shape community structure. Furthermore, we reveal a multidimensional stability mechanism, suggesting that the long‐term persistence of pioneer communities may rely on the functional complementarity between taxonomic redundancy and phylogenetic potential pool across time. At the ecological scale, bio9 acts as an environmental filter shaping taxonomic composition, whereas at the evolutionary scale, bio19 influences the regional species pool and thus determines phylogenetic structure. Finally, our analytical framework explicitly quantifies the inherent spatial effects within the SFTS approach, demonstrating that spatial structure plays an essential role in explaining temporal variation. Long‐term monitoring data are still necessary to uncover the ecological–evolutionary differentiation and mechanisms driving succession.

Overall, by linking ecological processes with evolutionary constraints, the general understanding of pine forest succession is enriched theoretically, as well as the sustainable management of pine forest ecosystems is advanced by stage‐specific strategies.

## Author Contributions

**Xiaofan Wang:** conceptualization (equal), data curation (equal), formal analysis (equal), investigation (equal), visualization (lead), writing – original draft (lead). **Yunfei Ma:** data curation (equal), investigation (equal). **Biao Zhao:** data curation (equal), investigation (equal). **Dengpeng Chen:** data curation (equal), investigation (equal). **Yehong Luo:** data curation (equal). **Mingchun Peng:** investigation (equal), writing – review and editing (equal). **Yongping Li:** investigation (equal), writing – review and editing (equal). **Xinmao Zhou:** investigation (equal), writing – review and editing (equal). **Wen Chen:** writing – review and editing (equal). **Cindy Q. Tang:** funding acquisition (equal), writing – review and editing (equal). **Chongyun Wang:** data curation (equal), funding acquisition (equal), investigation (equal), methodology (equal), writing – review and editing (equal).

## Funding

This study received financial support from the Major Program for Basic Research Project of Yunnan Province, China (202101bc070002), and the Special Foundation for National Science and Technology Basic Resources Investigation of China (2019FY202302).

## Conflicts of Interest

The authors declare no conflicts of interest.

## Supporting information


**Figure S1:** Relationships between field‐recorded disturbance levels and remote‐sensing‐based disturbance indicators.
**Figure S2:** Correlation between environmental factors and spatial factors.
**Figure S3:** Phylogenetic tree of all species in *Pinus kesiya* var. *langbianensis* forest (PKF).
**Figure S4:** Stand structural characteristics of PKF across five successional stages.
**Figure S5:** Variation trend of explanatory rate of spatial factors and environmental factors in PKF succession processes.
**Table S1:** Field investigation plots of *Pinus kesiya* var. *langbianensis* forest (PKF).
**Table S2:** 16 MEM principal component axes with significant positive eigenvalues.
**Table S3:** List of screened explanatory variables.
**Table S4:** Results of DCA ordination parameters.
**Table S5:** Mantel test of taxonomic β diversity (TβD) and phylogenetic β diversity (PβD) at different successional stages of PKF.

**Data S1:** ece372911‐sup‐0002‐DataS1.xlsx.

## Data Availability

The data that support the findings of this study are available in the Figures S1–S5 and Tables S1–S5, Data S1 of this article.

## References

[ece372911-bib-0001] Abbas, S. , J. E. Nichol , J. Zhang , and G. A. Fischer . 2019. “The Accumulation of Species and Recovery of Species Composition Along a 70 Year Succession in a Tropical Secondary Forest.” Ecological Indicators 106: 105524. 10.1016/j.ecolind.2019.105524.

[ece372911-bib-0002] Alberdi, I. , I. Cañellas , L. Hernández , and S. Condés . 2013. “A New Method for the Identification of Old‐Growth Trees in National Forest Inventories: Application to *Pinus halepensis* Mill. Stands in Spain.” Annals of Forest Science 70: 277–285. 10.1007/s13595-012-0261-9.

[ece372911-bib-0003] Anyomi, K. A. , B. Neary , J. Chen , and S. J. Mayor . 2022. “A Critical Review of Successional Dynamics in Boreal Forests of North America.” Environmental Reviews 30: 563–594. 10.1139/er-2021-0106.

[ece372911-bib-0004] Ashton, P. , and H. Zhu . 2020. “The Tropical‐Subtropical Evergreen Forest Transition in East Asia: An Exploration.” Plant Diversity 42: 255–280. 10.1016/j.pld.2020.04.001. 33094198 PMC7567766

[ece372911-bib-0005] Baldissera, T. C. , E. Frak , P. C. F. Carvalho , and G. Louarn . 2014. “Plant Development Controls Leaf Area Expansion in Alfalfa Plants Competing for Light.” Annals of Botany 113: 145–157. 10.1093/aob/mct251. 24201140 PMC3864722

[ece372911-bib-0007] Baniya, C. B. , T. Solhøy , and O. R. Vetaas . 2009. “Temporal Changes in Species Diversity and Composition in Abandoned Fields in a Trans‐Himalayan Landscape, Nepal.” Plant Ecology 201: 383–399. http://www.jstor.org/stable/40305646.

[ece372911-bib-0008] Bartlein, P. J. , S. Harrison , S. Brewer , et al. 2011. “Pollen‐Based Continental Climate Reconstructions at 6 and 21 Ka: A Global Synthesis.” Climate Dynamics 37: 775–802. 10.1007/s00382-010-0904-1.

[ece372911-bib-0009] Baselga, A. 2010. “Partitioning the Turnover and Nestedness Components of Beta Diversity.” Global Ecology and Biogeography 19: 134–143. 10.1111/j.1466-8238.2009.00490.x.

[ece372911-bib-0010] Bastow Wilson, J. 2012. “Species Presence/Absence Sometimes Represents a Plant Community as Well as Species Abundances Do, or Better.” Journal of Vegetation Science 23: 1013–1023. 10.1111/j.1654-1103.2012.01430.x.

[ece372911-bib-0011] Bazzaz, F. A. 1968. “Succession on Abandoned Fields in the Shawnee Hills, Southern Illinois.” Ecology 49: 924–936. 10.2307/1936544.

[ece372911-bib-0012] Bezemer, T. M. , and W. H. Van Der Putten . 2007. “Diversity and Stability in Plant Communities.” Nature 446: E6–E7. 10.1038/nature05749. 17392741

[ece372911-bib-0013] Bhaskar, R. , T. E. Dawson , and P. Balvanera . 2014. “Community Assembly and Functional Diversity Along Succession Post‐Management.” Functional Ecology 28: 1256–1265. 10.1111/1365-2435.12257.

[ece372911-bib-0014] Blois, J. L. , J. W. Williams , M. C. Fitzpatrick , and S. Ferrier . 2013. “Space Can Substitute for Time in Predicting Climate‐Change Effects on Biodiversity.” Proceedings of the National Academy of Sciences of the United States of America 110: 9374–9379. 10.1073/pnas.1220228110. 23690569 PMC3677423

[ece372911-bib-0015] Borcard, D. , F. Gillet , and P. Legendre . 2011. Numerical Ecology With R. Vol. 2, 688. Springer. 10.1007/978-1-4419-7976-6.

[ece372911-bib-0016] Borcard, D. , and P. Legendre . 2002. “All‐Scale Spatial Analysis of Ecological Data by Means of Principal Coordinates of Neighbour Matrices.” Ecological Modelling 153: 51–68. 10.1016/S0304-3800(01)00501-4.

[ece372911-bib-0017] Borcard, D. , P. Legendre , C. Avois‐Jacquet , and H. Tuomisto . 2004. “Dissecting the Spatial Structure of Ecological Data at Multiple Scales.” Ecology 85: 1826–1832. 10.1890/03-3111.

[ece372911-bib-0018] Bruelheide, H. , M. Böhnke , S. Both , et al. 2011. “Community Assembly During Secondary Forest Succession in a Chinese Subtropical Forest.” Ecological Monographs 81: 25–41. 10.1890/09-2172.1.

[ece372911-bib-0019] Cadotte, M. W. , R. Dinnage , and D. Tilman . 2012. “Phylogenetic Diversity Promotes Ecosystem Stability.” Ecology 93: S223. 10.1890/11-0426.1.

[ece372911-bib-0020] Cadotte, M. W. , and C. M. Tucker . 2017. “Should Environmental Filtering Be Abandoned?” Trends in Ecology and Evolution 32: 429–437. 10.1016/j.tree.2017.03.004. 28363350

[ece372911-bib-0021] Cavender‐Bares, J. , D. D. Ackerly , D. A. Baum , F. A. Bazzaz , and S. S. Renner . 2004. “Phylogenetic Overdispersion in Floridian Oak Communities.” American Naturalist 163: 823–843. 10.1086/386375. 15266381

[ece372911-bib-0022] Cavender‐Bares, J. , A. Keen , and B. Miles . 2006. “Phylogenetic Structure of Floridian Plant Communities Depends on Taxonomic and Spatial Scale.” Ecology 87: S109–S122. 10.1890/0012-9658(2006)87[109:PSOFPC]2.0.CO;2. 16922307

[ece372911-bib-0023] Cavender‐Bares, J. , K. H. Kozak , P. V. A. Fine , and S. W. Kembel . 2009. “The Merging of Community Ecology and Phylogenetic Biology.” Ecology Letters 12: 693–715. 10.1111/j.1461-0248.2009.01314.x. 19473217

[ece372911-bib-0024] Chai, P. T. , J. J. Xie , L. S. Yang , et al. 2023. “Community Vertical Stratification Drives Temporal Taxonomic and Phylogenetic Beta Diversity in a Mixed Broadleaf‐Conifer Forest.” Frontiers in Ecology and Evolution 11: 1170197. 10.3389/fevo.2023.1170197.

[ece372911-bib-0025] Chai, Y. F. , M. Yue , X. Liu , et al. 2016. “Patterns of Taxonomic, Phylogenetic Diversity During a Long‐Term Succession of Forest on the Loess Plateau, China: Insights Into Assembly Process.” Scientific Reports 6: 27087. 10.1038/srep27087. 27272407 PMC4897607

[ece372911-bib-0026] Chase, J. M. 2007. “Drought Mediates the Importance of Stochastic Community Assembly.” Proceedings of the National Academy of Sciences of the United States of America 104: 17430–17434. 10.1073/pnas.0704350104. 17942690 PMC2077273

[ece372911-bib-0027] Chave, J. 2004. “Neutral Theory and Community Ecology.” Ecology Letters 7: 241–253. 10.1111/j.1461-0248.2003.00566.x.

[ece372911-bib-0028] Chen, J. , and O. T. Lewis . 2024. “Limits to Species Distributions on Tropical Mountains Shift From High Temperature to Competition as Elevation Increases.” Ecological Monographs 94: e1597. 10.1002/ecm.1597.

[ece372911-bib-0029] Cowles, H. C. 1899. “The Ecological Relations of the Vegetation on the Sand Dunes of Lake Michigan.” Botanical Gazette 27: 361–391. https://www.jstor.org/stable/2464960.

[ece372911-bib-0030] Crouzeilles, R. , M. Curran , M. S. Ferreira , D. B. Lindenmayer , C. E. Grelle , and J. M. Rey Benayas . 2016. “A Global Meta‐Analysis on the Ecological Drivers of Forest Restoration Success.” Nature Communications 7: 11666. 10.1038/ncomms11666. PMC4874030 27193756

[ece372911-bib-0031] da Costa, J. S. , F. A. Carvalho , L. E. Fernandes , et al. 2025. “Unraveling Niche Complementarity and Mass Ratio Hypotheses Along Amazon Forest Succession: Functional Composition a Key Factor for Restoration.” Acta Oecologica 127: 104083. 10.1016/j.actao.2025.104083.

[ece372911-bib-0032] Diamond, J. M. 1975. “Assembly of Species Communities.” In Ecology and Evolution of Communities, edited by M. L. Cody and J. M. Diamond , 342–444. Harvard University Press.

[ece372911-bib-0033] Duan, J. H. , L. Yang , T. Tang , et al. 2024. “Environment and Management Jointly Shape the Spatial Patterns of Plant Species Diversity of Moist Grasslands in the Mountains of Northeastern Yunnan.” Plant Diversity 46: 744–754. 10.1016/j.pld.2024.04.005. 39811816 PMC11726043

[ece372911-bib-0034] Eckert, A. J. , and B. D. Hall . 2006. “Phylogeny, Historical Biogeography, and Patterns of Diversification for Pinus (Pinaceae): Phylogenetic Tests of Fossil‐Based Hypotheses.” Molecular Phylogenetics and Evolution 40: 166–182. 10.1016/j.ympev.2006.03.009. 16621612

[ece372911-bib-0035] Emerson, B. C. , and R. G. Gillespie . 2008. “Phylogenetic Analysis of Community Assembly and Structure Over Space and Time.” Trends in Ecology and Evolution 23: 619–630. 10.1016/j.tree.2008.07.005. 18823678

[ece372911-bib-0036] Faith, D. P. 1992. “Conservation Evaluation and Phylogenetic Diversity.” Biological Conservation 61: 1–10. 10.1016/0006-3207(92)91201-3.

[ece372911-bib-0037] Fan, Q. L. , H. Xu , D. P. Luo , et al. 2024. “Characterising Spatial Effects of Individual Tree and Component Biomass for Three Typical Tree Species in Yunnan, China.” Ecological Indicators 159: 111705. 10.1016/j.ecolind.2024.111705.

[ece372911-bib-0038] Fickert, T. , and F. Grüninger . 2018. “High‐Speed Colonization of Bare Ground—Permanent Plot Studies on Primary Succession of Plants in Recently Deglaciated Glacier Forelands.” Land Degradation and Development 29: 2668–2680. 10.1002/ldr.3063.

[ece372911-bib-0039] Foster, B. L. , and D. Tilman . 2000. “Dynamic and Static Views of Succession: Testing the Descriptive Power of the Chronosequence Approach.” Plant Ecology 146: 1–10. 10.1023/A:1009895103017.

[ece372911-bib-0040] Fu, R. Y. , L. C. Dai , Z. H. Zhang , and G. Hu . 2023. “Community Assembly Along a Successional Chronosequence in the Northern Tropical Karst Mountains, South China.” Plant and Soil 491: 317–331. 10.1007/s11104-023-06118-z.

[ece372911-bib-0041] Fukami, T. , and D. A. Wardle . 2005. “Long‐Term Ecological Dynamics: Reciprocal Insights From Natural and Anthropogenic Gradients.” Proceedings of the Royal Society B: Biological Sciences 272: 2105. 10.1098/rspb.2005.3277. PMC1559953 16191623

[ece372911-bib-0042] Gao, Z. L. , J. H. Wei , Z. Li , et al. 2021. “Effect of Slope on Soil Carbon Storage in Prescribed Burning–A Case Study of Pinus Kesiya in Jinggu County Yunnan Province.” IOP Conference Series: Earth and Environmental Science 631: 012042. 10.1088/1755-1315/631/1/012042.

[ece372911-bib-0043] Geng, Q. W. , A. Muhammad , Z. X. Yuan , et al. 2022. “Plant Species Composition and Diversity Along Successional Gradients in Arid and Semi‐Arid Regions of China.” Forest Ecology and Management 524: 120542. 10.1016/j.foreco.2022.120542.

[ece372911-bib-0044] Graham, C. H. , and P. V. A. Fine . 2008. “Phylogenetic Beta Diversity: Linking Ecological and Evolutionary Processes Across Space in Time.” Ecology Letters 11: 1265–1277. 10.1111/j.1461-0248.2008.01256.x. 19046358

[ece372911-bib-0045] Gravel, D. , C. D. Canham , M. Beaudet , and C. Messier . 2006. “Reconciling Niche and Neutrality: The Continuum Hypothesis.” Ecology Letters 9: 399–409. 10.1111/j.1461-0248.2006.00884.x. 16623725

[ece372911-bib-0046] Gu, Z. J. , and M. X. Li . 1982. “A Study on the Karyotype in *Pinus yunnanensis* and *Pinus kesiya* Var. *langbianensis* .” Plant Diversity 4: 1–3.

[ece372911-bib-0047] Guénard, G. , and P. Legendre . 2022. “Hierarchical Clustering With Contiguity Constraint in R.” Journal of Statistical Software 103: 1–26. 10.18637/jss.v103.i07.

[ece372911-bib-0049] He, R. , M. Hu , H. Shi , et al. 2022. “Patterns of Species Diversity and Its Determinants in a Temperate Deciduous Broad‐Leaved Forest.” Forest Ecosystems 9: 100062. 10.1016/j.fecs.2022.100062.

[ece372911-bib-0050] HilleRisLambers, J. , P. B. Adler , W. S. Harpole , J. M. Levine , and M. M. Mayfield . 2012. “Rethinking Community Assembly Through the Lens of Coexistence Theory.” Annual Review of Ecology, Evolution, and Systematics 43: 227–248. 10.1146/annurev-ecolsys-110411-160411.

[ece372911-bib-0051] Hoffmeister, T. S. , L. E. M. Vet , A. Biere , K. Holsinger , and J. Filser . 2005. “Ecological and Evolutionary Consequences of Biological Invasion and Habitat Fragmentation.” Ecosystems 8: 657–667. 10.1007/s10021-003-0138-8.

[ece372911-bib-0052] Hou, M. M. , X. Y. Li , J. W. Wang , S. Liu , and X. H. Zhao . 2017. “Phylogenetic Development and Functional Structures During Successional Stages of Conifer and Broad‐Leaved Mixed Forest Communities in Changbai Mountains, China.” Acta Ecologica Sinica 37: 7503–7512. 10.5846/stxb201609141860.

[ece372911-bib-0053] Howard, L. F. , and T. D. Lee . 2003. “Temporal Patterns of Vascular Plant Diversity in Southeastern New Hampshire Forests.” Forest Ecology and Management 185: 5–20. 10.1016/S0378-1127(03)00243-3.

[ece372911-bib-0054] Hubbell, S. P. 2011. The Unified Neutral Theory of Biodiversity and Biogeography (MPB‐32). Princeton University Press. 10.1515/9781400837526. 21561679

[ece372911-bib-0055] Isbell, F. I. , H. W. Polley , and B. J. Wilsey . 2009. “Biodiversity, Productivity and the Temporal Stability of Productivity: Patterns and Processes.” Ecology Letters 12: 443–451. 10.1111/j.1461-0248.2009.01299.x. 19379138

[ece372911-bib-0056] Janečková, P. , L. Tichý , L. R. Walker , and K. Prach . 2024. “Global Drivers Influencing Vegetation During Succession: Factors and Implications.” Journal of Vegetation Science 35: e13297. 10.1111/jvs.13297.

[ece372911-bib-0057] Jarzyna, M. A. , and W. Jetz . 2016. “Detecting the Multiple Facets of Biodiversity.” Trends in Ecology & Evolution 31: 527–538. 10.1016/j.tree.2016.04.002. 27168115

[ece372911-bib-0058] Jarzyna, M. A. , I. Quintero , and W. Jetz . 2021. “Global Functional and Phylogenetic Structure of Avian Assemblages Across Elevation and Latitude.” Ecology Letters 24: 196–207. 10.1111/ele.13631. 33124188

[ece372911-bib-0059] Jiang, Y. D. , S. G. Li , and Y. C. Liu . 2017. “Review on the Research Progress of the Oriented Cultivation Techniques for the High‐Resin‐Yield *Pinus Kesiya* Var. *Langbianensis* .” Journal of West China Forestry Science 46: 32–37. 10.16473/j.cnki.xblykx1972.2017.02.006.

[ece372911-bib-0060] Jin, L. S. , M. W. Cadotte , and M. J. Fortin . 2015. “Phylogenetic Turnover Patterns Consistent With Niche Conservatism in Montane Plant Species.” Journal of Ecology 103: 742–749. 10.1111/1365-2745.12385.

[ece372911-bib-0061] Jin, Y. , and H. Qian . 2019. “V. PhyloMaker: An R Package That Can Generate Very Large Phylogenies for Vascular Plants.” Ecography 42: 1353–1359. 10.1111/ecog.04434. PMC9363651 35967255

[ece372911-bib-0062] Johnson, E. A. , and K. Miyanishi . 2008. “Testing the Assumptions of Chronosequences in Succession.” Ecology Letters 11: 419–431. 10.1111/j.1461-0248.2008.01173.x. 18341585

[ece372911-bib-0063] Karimi, N. , D. J. Larkin , M. C. Glasenhardt , et al. 2022. “Selection on Convergent Functional Traits Drives Compositional Divergence in Early Succession of a Tallgrass Prairie Restoration Experiment.” Journal of Ecology 110: 415–429. 10.1111/1365-2745.13808.

[ece372911-bib-0064] Kassen, R. , A. Buckling , G. Bell , and P. B. Rainey . 2000. “Diversity Peaks at Intermediate Productivity in a Laboratory Microcosm.” Nature 406: 508–512. 10.1038/35020060. 10952310

[ece372911-bib-0065] Kembel, S. W. , P. D. Cowan , M. R. Helmus , et al. 2010. “Picante: R Tools for Integrating Phylogenies and Ecology.” Bioinformatics 26: 1463–1464. 10.1093/bioinformatics/btq166. 20395285

[ece372911-bib-0066] Kharouba, H. M. , and J. L. Williams . 2024. “Forecasting Species' Responses to Climate Change Using Space‐For‐Time Substitution.” Trends in Ecology and Evolution 39: 716–725. 10.1016/j.tree.2024.03.009. 38744627

[ece372911-bib-0067] Kong, F. H. , X. R. Chen , M. H. Zhang , et al. 2023. “Pioneer Tree Species Accumulate Higher Neighbourhood Diversity Than Late‐Successional Species in a Subtropical Forest.” Forest Ecology and Management 531: 120740. 10.1016/j.foreco.2022.120740.

[ece372911-bib-0068] Kraft, N. J. B. , R. Valencia , and D. D. Ackerly . 2008. “Functional Traits and Niche‐Based Tree Community Assembly in an Amazonian Forest.” Science 322: 580–582. 10.1126/science.1160662. 18948539

[ece372911-bib-0069] Lai, J. S. , Y. Zou , J. L. Zhang , and P. R. Peres‐Neto . 2022. “Generalizing Hierarchical and Variation Partitioning in Multiple Regression and Canonical Analyses Using the Rdacca. Hp R Package.” Methods in Ecology and Evolution 13: 782–788. 10.1111/2041-210X.13800.

[ece372911-bib-0070] Lanta, V. , O. Mudrák , M. Dvorský , et al. 2023. “Multifaceted Diversity Changes Reveal Community Assembly Mechanisms During Early Stages of Post‐Logging Forest Succession.” Plant Ecology 224: 335–347. 10.1007/s11258-023-01306-4.

[ece372911-bib-0071] Lanta, V. , P. Sebek , P. Kozel , et al. 2025. “Plant and Saproxylic Beetle Dynamics During Succession in Lowland Temperate Broadleaf Forests Reveal Only Short Periods of Increased Diversity.” Biological Conservation 308: 111258. 10.1016/j.biocon.2025.111258.

[ece372911-bib-0072] Lasky, J. R. , M. Uriarte , V. K. Boukili , and R. L. Chazdon . 2014. “Trait‐Mediated Assembly Processes Predict Successional Changes in Community Diversity of Tropical Forests.” Proceedings of the National Academy of Sciences of the United States of America 111: 5616–5621. 10.1073/pnas.1319342111. 24706791 PMC3992673

[ece372911-bib-0073] Lazzaro, L. , L. Lastrucci , D. Viciani , R. Benesperi , V. Gonnelli , and A. Coppi . 2020. “Patterns of Change in α and β Taxonomic and Phylogenetic Diversity in the Secondary Succession of Semi‐Natural Grasslands in the Northern Apennines.” PeerJ 8: e8683. 10.7717/peerj.8683. 32201641 PMC7071822

[ece372911-bib-0074] Lebrija‐Trejos, E. , E. A. Pérez‐García , J. A. Meave , F. Bongers , and L. Poorter . 2010. “Functional Traits and Environmental Filtering Drive Community Assembly in a Species‐Rich Tropical System.” Ecology 91: 386–398. 10.1890/08-1449.1. 20392004

[ece372911-bib-0075] Lebrija‐Trejos, E. , P. B. Reich , A. Hernández , and S. J. Wright . 2016. “Species With Greater Seed Mass Are More Tolerant of Conspecific Neighbours: A Key Driver of Early Survival and Future Abundances in a Tropical Forest.” Ecology Letters 19: 1071–1080. 10.1111/ele.12643. 27346439

[ece372911-bib-0077] Legendre, P. 1993. “Spatial Autocorrelation: Trouble or New Paradigm?” Ecology 74: 1659–1673. 10.2307/1939924.

[ece372911-bib-0076] Legendre, P. , X. C. Mi , H. B. Ren , et al. 2009. “Partitioning Beta Diversity in a Subtropical Broad‐Leaved Forest of China.” Ecology 90: 663–674. 10.1890/07-1880.1. 19341137

[ece372911-bib-0079] Letcher, S. G. 2010. “Phylogenetic Structure of Angiosperm Communities During Tropical Forest Succession.” Proceedings of the Royal Society B: Biological Sciences 277: 97–104. 10.1098/rspb.2009.0865. PMC2842617 19801375

[ece372911-bib-0078] Letcher, S. G. , R. L. Chazdon , A. C. S. Andrade , et al. 2012. “Phylogenetic Community Structure During Succession: Evidence From Three Neotropical Forest Sites.” Perspectives in Plant Ecology, Evolution and Systematics 14: 79–87. 10.1016/j.ppees.2011.09.005.

[ece372911-bib-0080] Letten, A. D. , D. A. Keith , and M. G. Tozer . 2014. “Phylogenetic and Functional Dissimilarity Does Not Increase During Temporal Heathland Succession.” Proceedings of the Royal Society B: Biological Sciences 281: 20142102. 10.1098/rspb.2014.2102. PMC4240997 25377459

[ece372911-bib-0081] Li, J. 2010. Dynamics of Biomass and Carbon Stock for Young and Middle Aged Plantation of Simao Pine (Pinus kesiya var. langbianensis). Beijing Forestry University.

[ece372911-bib-0082] Li, S. F. , X. D. Lang , W. D. Liu , G. L. Ou , H. Xu , and J. R. Su . 2018. “The Relationship Between Species Richness and Aboveground Biomass in a Primary *Pinus kesiya* Forest of Yunnan, Southwestern China.” PLoS One 13: e0191140. 10.1371/journal.pone.0191140. 29324901 PMC5764369

[ece372911-bib-0083] Li, S. F. , J. R. Su , W. D. Liu , et al. 2013. “Quantitative Classification of *Pinus kesiya* Var. *langbianensis* Communities and Their Species Richness in Relation to the Environmental Factors in Yunnan Province of Southwest China.” Chinese Journal of Ecology 32: 3152–3519. 10.13292/j.1000-4890.2013.0484.

[ece372911-bib-0084] Li, S. P. , M. W. Cadotte , S. J. Meiners , Z. S. Hua , L. Jiang , and W. S. Shu . 2015. “Species Colonisation, Not Competitive Exclusion, Drives Community Overdispersion Over Long‐Term Succession.” Ecology Letters 18: 964–973. 10.1111/ele.12476. 26189648

[ece372911-bib-0085] Li, S. P. , M. W. Cadotte , S. J. Meiners , Z. Pu , T. Fukami , and L. Jiang . 2016. “Convergence and Divergence in a Long‐Term Old‐Field Succession: The Importance of Spatial Scale and Species Abundance.” Ecology Letters 19: 1101–1109. 10.1111/ele.12647. 27373449

[ece372911-bib-0086] Li, T. X. , L. Xu , F. Wang , et al. 2022. “Novel Evidence From *Taxus fuana* Forests for Niche‐Neutral Process Assembling Community.” Forest Ecosystems 9: 100035. 10.1016/j.fecs.2022.100035.

[ece372911-bib-0087] Li, Y. Y. , M. C. Peng , and C. L. Dang . 2013. “A Study on the Gap Generation of *Pinus kesiya* Var. *langbianensis* in Weiyuanjiang Nature Reserve.” Environmental Science and Surveys 32: 20–25. 10.13623/j.cnki.hkdk.2013.02.001.

[ece372911-bib-0088] Liu, C. , T. T. Sun , X. Wu , L. Tan , Q. H. Cai , and T. Tang . 2023. “Disentangling Multiple Relationships of Species Diversity, Functional Diversity, Diatom Community Biomass and Environmental Variables in a Mountainous Watershed.” Frontiers in Ecology and Evolution 11: 1150001. 10.3389/fevo.2023.1150001.

[ece372911-bib-0089] Liu, C. X. , Y. Wu , X. L. Zhang , et al. 2024. “Response of Individual‐Tree Aboveground Biomass to Spatial Effects in *Pinus kesiya* Var. *langbianensis* Forests by Stand Origin and Tree Size.” Forests 15: 349. 10.3390/f15020349.

[ece372911-bib-0090] Liu, J. L. , H. Qian , Y. Jin , et al. 2016. “Disentangling the Drivers of Taxonomic and Phylogenetic Beta Diversities in Disturbed and Undisturbed Subtropical Forests.” Scientific Reports 6: 35926. 10.1038/srep35926. 27775021 PMC5075936

[ece372911-bib-0091] Liu, M. X. , Y. B. Ma , X. Y. Wang , and L. Xu . 2021. “Plant Community Assembly Mechanisms of a Subalpine Meadow Community Along Different Successional Time.” Rangeland Ecology & Management 77: 118–125. 10.1016/j.rama.2021.04.006.

[ece372911-bib-0092] Liu, Y. , M. Zhang , W. Q. Peng , et al. 2021. “Phylogenetic and Functional Diversity Could Be Better Indicators of Macroinvertebrate Community Stability.” Ecological Indicators 129: 107892. 10.1016/j.ecolind.2021.107892.

[ece372911-bib-0093] Liu, Y. F. , Y. Z. Wang , Z. M. Song , et al. 2022. “A New Diterpenoid Acid From the Rosin of *Pinus kesiya* Var. *langbianensis* (A. Chev.) Gaussen ex Bui.” Acta Pharmaceutica Sinica 57: 2786–2790.

[ece372911-bib-0094] Lohbeck, M. , L. Poorter , E. Lebrija‐Trejos , et al. 2013. “Successional Changes in Functional Composition Contrast for Dry and Wet Tropical Forest.” Ecology 94: 1211–1216. 10.1890/12-1850.1. 23923479

[ece372911-bib-0095] Lohbeck, M. , L. Poorter , M. Martínez‐Ramos , J. Rodriguez‐Velázquez , M. van Breugel , and F. Bongers . 2014. “Changing Drivers of Species Dominance During Tropical Forest Succession.” Functional Ecology 28: 1052–1058. 10.1111/1365-2435.12240.

[ece372911-bib-0096] Luo, Y. S. , M. L. Zhou , S. S. Jin , Q. X. Wang , and D. F. Yan . 2023. “Changes in Phylogenetic Structure and Species Composition of Woody Plant Communities Across an Elevational Gradient in the Southern Taihang Mountains, China.” Global Ecology and Conservation 42: e02412. 10.1016/j.gecco.2023.e02412.

[ece372911-bib-0097] MacArthur, R. 1955. “Fluctuations of Animal Populations and a Measure of Community Stability.” Ecology 36: 533–536. 10.2307/1929601.

[ece372911-bib-0098] MacArthur, R. , and R. Levins . 1967. “The Limiting Similarity, Convergence, and Divergence of Coexisting Species.” American Naturalist 101: 377–385. 10.1086/282505.

[ece372911-bib-0099] Maleki, K. , M. A. Gueye , B. Lafleur , A. Leduc , and Y. Bergeron . 2019. “Modelling Post‐Disturbance Successional Dynamics of the Canadian Boreal Mixedwoods.” Forests 11: 3. 10.3390/f11010003.

[ece372911-bib-0100] Marcilio‐Silva, V. , V. D. Pillar , and M. C. M. Marques . 2016. “Functional Turnover and Community Assemblage During Tropical Forest Succession.” Community Ecology 17: 88–97. 10.1556/168.2016.17.1.11.

[ece372911-bib-0101] Martínez‐Ramos, M. , F. Barragán , F. Mora , et al. 2021. “Differential Ecological Filtering Across Life Cycle Stages Drive Old‐Field Succession in a Neotropical Dry Forest.” Forest Ecology and Management 482: 118810. 10.1016/j.foreco.2020.118810.

[ece372911-bib-0102] Mastrogianni, A. , D. A. Kiziridis , M. Pleniou , F. Xystrakis , S. Tsiftsis , and I. Tsiripidis . 2024. “Imprints of Land Use History and Disturbance Regime in Phylogenetic Diversity of Mediterranean Plant Communities.” Ecosphere 15: e4972. 10.1002/ecs2.4972.

[ece372911-bib-0103] Mayfield, M. M. , and J. M. Levine . 2010. “Opposing Effects of Competitive Exclusion on the Phylogenetic Structure of Communities.” Ecology Letters 13: 1085–1093. 10.1111/j.1461-0248.2010.01509.x. 20576030

[ece372911-bib-0104] McKone, M. J. , E. W. Williams , and J. J. Beck . 2021. “Evidence for Local‐Scale Community Assembly Processes From Long‐Term Observations of Biodiversity in a Grassland Chronosequence.” Journal of Vegetation Science 32: e13065. 10.1111/jvs.13065.

[ece372911-bib-0105] Meiners, S. J. , M. W. Cadotte , J. D. Fridley , S. T. A. Pickett , and L. R. Walker . 2015. “Is Successional Research Nearing Its Climax? New Approaches for Understanding Dynamic Communities.” Functional Ecology 29: 154–164. 10.1111/1365-2435.12391.

[ece372911-bib-0106] Mi, X. C. , N. G. Swenson , Q. Jia , et al. 2016. “Stochastic Assembly in a Subtropical Forest Chronosequence: Evidence From Contrasting Changes of Species, Phylogenetic and Functional Dissimilarity Over Succession.” Scientific Reports 6: 32596. 10.1038/srep32596. 27599883 PMC5013490

[ece372911-bib-0107] Miao, R. H. , X. L. Qiu , M. X. Guo , A. Musa , and D. M. Jiang . 2018. “Accuracy of Space‐For‐Time Substitution for Vegetation State Prediction Following Shrub Restoration.” Journal of Plant Ecology 11: 208–217. 10.1093/jpe/rtw133.

[ece372911-bib-0108] Mo, X. X. , L. L. Shi , Y. J. Zhang , H. Zhu , and J. F. Slik . 2013. “Change in Phylogenetic Community Structure During Succession of Traditionally Managed Tropical Rainforest in Southwest China.” PLoS One 8: e71464. 10.1371/journal.pone.0071464. 23936268 PMC3729948

[ece372911-bib-0109] Muscarella, R. , M. Uriarte , T. M. Aide , et al. 2016. “Functional Convergence and Phylogenetic Divergence During Secondary Succession of Subtropical Wet Forests in Puerto Rico.” Journal of Vegetation Science 27: 283–294. 10.1111/jvs.12354.

[ece372911-bib-0110] Myers, J. A. , and K. E. Harms . 2009. “Seed Arrival, Ecological Filters, and Plant Species Richness: A Meta‐Analysis.” Ecology Letters 12: 1250–1260. 10.1111/j.1461-0248.2009.01373.x. 19723285

[ece372911-bib-0111] Naeem, S. , and S. B. Li . 1997. “Biodiversity Enhances Ecosystem Reliability.” Nature 390: 507–509. 10.1038/37348.

[ece372911-bib-0113] Niu, K. C. , Y. N. Liu , Z. H. Shen , F. L. He , and J. Y. Fang . 2009. “Community Assembly: The Relative Importance of Neutral Theory and Niche Theory.” Biodiversity Science 17: 579–593. 10.3724/SP.J.1003.2009.09142.

[ece372911-bib-0114] Norden, N. , R. L. Chazdon , A. Chao , Y. H. Jiang , and B. Vilchez‐Alvarado . 2009. “Resilience of Tropical Rain Forests: Tree Community Reassembly in Secondary Forests.” Ecology Letters 12: 385–394. 10.1111/j.1461-0248.2009.01292.x. 19379133

[ece372911-bib-0115] Norden, N. , S. G. Letcher , V. Boulili , N. G. Swenson , and R. Chazdon . 2012. “Demographic Drivers of Successional Changes in Phylogenetic Structure Across Life‐History Stages in Plant Communities.” Ecology 93: S70–S82. 10.1890/10-2179.1.

[ece372911-bib-0116] Odum, E. P. 1983. Basic Ecology. Saunders College Publishing.

[ece372911-bib-0117] Oksanen, J. , F. G. Blanchet , M. Friendly , et al. 2019. “vegan: Community Ecology Package. R Package Version 2.5‐6.” https://cran.r‐project.org/web/packages/vegan.

[ece372911-bib-0118] Oliver, C. D. , and B. C. Larson . 1996. Forest Stand Dynamics. John Wiley & Sons.

[ece372911-bib-0119] Omidipour, R. , P. Tahmasebi , M. F. Faizabadi , M. Faramarzi , and A. Ebrahimi . 2021. “Does β Diversity Predict Ecosystem Productivity Better Than Species Diversity?” Ecological Indicators 122: 107212. 10.1016/j.ecolind.2020.107212.

[ece372911-bib-0120] Ouyang, S. , W. H. Xiang , X. P. Wang , et al. 2016. “Significant Effects of Biodiversity on Forest Biomass During the Succession of Subtropical Forest in South China.” Forest Ecology and Management 372: 291–302. 10.1016/j.foreco.2016.04.020.

[ece372911-bib-0121] Parrish, J. A. D. , and F. A. Bazzaz . 1982. “Competitive Interactions in Plant Communities of Different Successional Ages.” Ecology 63: 314–320. 10.2307/1938948.

[ece372911-bib-0122] Pausas, J. G. , and M. Verdu . 2010. “The Jungle of Methods for Evaluating Phenotypic and Phylogenetic Structure of Communities.” Bioscience 60: 614–625. 10.1525/bio.2010.60.8.7.

[ece372911-bib-0123] Pickett, S. T. A. 1989. “Space‐For‐Time Substitution as an Alternative to Long‐Term Studies.” In Long‐Term Studies in Ecology: Approaches and Alternatives, 110–135. Springer New York. 10.1007/978-1-4615-7358-6_5.

[ece372911-bib-0124] Poorter, L. , K. Kitajima , P. Mercado , J. Chubiña , I. Melgar , and H. H. Prins . 2010. “Resprouting as a Persistence Strategy of Tropical Forest Trees: Relations With Carbohydrate Storage and Shade Tolerance.” Ecology 91: 2613–2627. 10.1890/09-0862.1. 20957956

[ece372911-bib-0125] Poorter, L. , M. T. van Der Sande , L. Amissah , et al. 2024. “A Comprehensive Framework for Vegetation Succession.” Ecosphere 15: e4794. 10.1002/ecs2.4794.

[ece372911-bib-0126] Prach, K. , and L. R. Walker . 2011. “Four Opportunities for Studies of Ecological Succession.” Trends in Ecology and Evolution 26: 119–123. 10.1016/j.tree.2010.12.007. 21295370

[ece372911-bib-0127] Purschke, O. , S. G. Michalski , H. Bruelheide , and W. Durka . 2017. “Phylogenetic Turnover During Subtropical Forest Succession Across Environmental and Phylogenetic Scales.” Ecology and Evolution 7: 11079–11091. 10.1002/ece3.3564. 29299283 PMC5743486

[ece372911-bib-0128] Purschke, O. , B. C. Schmid , M. T. Sykes , et al. 2013. “Contrasting Changes in Taxonomic, Phylogenetic and Functional Diversity During a Long‐Term Succession: Insights Into Assembly Processes.” Journal of Ecology 101: 857–866. 10.1111/1365-2745.12098.

[ece372911-bib-0192] R Core Team . 2024. “R: A Language and Environment for Statistical Computing.” R Foundation for Statistical Computing. https://www.R‐project.org/.

[ece372911-bib-0129] Rolo, V. , P. I. Olivier , R. A. Gulburnond , and R. J. van Aarde . 2016. “Validating Space‐For‐Time Substitution in a New‐Growth Coastal Dune Forest.” Applied Vegetation Science 19: 235–243. 10.1111/avsc.12210.

[ece372911-bib-0130] Romanowski, L. L. , T. B. Zanata , M. C. M. Marques , M. B. Carlucci , and I. G. Varassin . 2021. “Increased Reproductive Trait Diversity, Evolutionary History and Distinctiveness During the Succession of Tropical Forest.” Journal of Vegetation Science 32: e13090. 10.1111/jvs.13090.

[ece372911-bib-0131] Sadeghinia, M. , M. Kargar , and Z. Jafarian . 2023. “The Relationship Between the Functional Diversity, Functional Redundancy and Community Stability in Mountain Rangelands.” Community Ecology 24: 1–8. 10.1007/s42974-022-00128-0.

[ece372911-bib-0132] Sánchez‐González, A. , and L. López‐Mata . 2005. “Plant Species Richness and Diversity Along an Altitudinal Gradient in the Sierra Nevada, Mexico.” Diversity and Distributions 11: 567–575. 10.1111/j.1366-9516.2005.00186.x.

[ece372911-bib-0133] Santos, B. A. , M. Tabarelli , F. P. L. Melo , et al. 2014. “Phylogenetic Impoverishment of Amazonian Tree Communities in an Experimentally Fragmented Forest Landscape.” PLoS One 9: e113109. 10.1371/journal.pone.0113109. 25409011 PMC4237388

[ece372911-bib-0134] Selaya, N. G. , and N. P. R. Anten . 2010. “Leaves of Pioneer and Later‐Successional Trees Have Similar Lifetime Carbon Gain in Tropical Secondary Forest.” Ecology 91: 1102–1113. 10.1890/08-2111.1. 20462124

[ece372911-bib-0136] Simpson, E. H. 1949. “Measurement of Diversity.” Nature 163: 688. 10.1038/163688a0.

[ece372911-bib-0137] Smale, M. C. , D. A. Coomes , R. L. Parfitt , D. A. Peltzer , N. W. H. Mason , and N. B. Fitzgerald . 2016. “Post‐Volcanic Forest Succession on New Zealand's North Island: An Appraisal From Long‐Term Plot Data.” New Zealand Journal of Botany 54: 11–29. 10.1080/0028825X.2015.1102747.

[ece372911-bib-0138] Song, L. , W. Y. Liu , W. Z. Ma , X. J. Zhao , M. Zhou , and G. P. Yang . 2011. “Community Characteristics of Monsoon Evergreen Broad‐Leaved and *Pinus kesiya* Var. *Langbianensis* Forests in the West Foot of Ailao Mountain, Yunnan.” Journal of Mountain Science 29: 164–172. 10.3724/SP.J.1011.2011.00353.

[ece372911-bib-0139] Song, M. , W. X. Peng , H. Du , and Q. G. Xu . 2019. “Responses of Soil and Microbial C: N: P Stoichiometry to Vegetation Succession in a Karst Region of Southwest China.” Forests 10: 755. 10.3390/f10090755.

[ece372911-bib-0140] Sousa, W. P. 1984. “The Role of Disturbance in Natural Communities.” Annual Review of Ecology, Evolution, and Systematics 15: 353–391. https://www.jstor.org/stable/2096953.

[ece372911-bib-0141] Spellerberg, I. F. , and P. J. Fedor . 2003. “A Tribute to Claude Shannon (1916–2001) and a Plea for More Rigorous Use of Species Richness, Species Diversity and the ‘Shannon‐Wiener’ Index.” Global Ecology and Biogeography 12: 177–179. 10.1046/j.1466-822X.2003.00015.x.

[ece372911-bib-0142] Spurr, S. H. 1952. “Origin of the Concept of Forest Succession.” Ecology 33: 426–427. 10.2307/1932839.

[ece372911-bib-0143] Stadler, J. , S. Klotz , R. Brandl , and S. Knapp . 2017. “Species Richness and Phylogenetic Structure in Plant Communities: 20 Years of Succession.” Web Ecology 17: 37–46. 10.5194/we-17-37-2017.

[ece372911-bib-0144] Stegen, J. C. , X. Lin , A. E. Konopka , and J. K. Fredrickson . 2012. “Stochastic and Deterministic Assembly Processes in Subsurface Microbial Communities.” ISME Journal 6: 1653–1664. 10.1038/ismej.2012.22. 22456445 PMC3498916

[ece372911-bib-0145] Stephenson, N. L. , and P. J. van Mantgem . 2005. “Forest Turnover Rates Follow Global and Regional Patterns of Productivity.” Ecology Letters 8: 524–531. 10.1111/j.1461-0248.2005.00746.x. 21352456

[ece372911-bib-0146] Swenson, N. G. , B. J. Enquist , J. Pither , J. Thompson , and J. K. Zimmerman . 2006. “The Problem and Promise of Scale Dependency in Community Phylogenetics.” Ecology 87: 2418–2424. 10.1890/0012-9658(2006)87[2418:TPAPOS]2.0.CO;2. 17089650

[ece372911-bib-0147] Symstad, A. J. , F. S. Chapin , D. H. Wall , et al. 2003. “Long‐Term and Large‐Scale Perspectives on the Relationship Between Biodiversity and Ecosystem Functioning.” Bioscience 53: 89–98. 10.1641/0006-3568(2003)053[0089:LTALSP]2.0.CO;2.

[ece372911-bib-0148] Tan, S. S. , Z. L. Ye , L. B. Yuan , et al. 2013. “Beta Diversity of Plant Communities in Baishanzu Nature Reserve.” Acta Ecologica Sinica 33: 6944–6956. 10.5846/stxb201207010920.

[ece372911-bib-0149] Tang, C. Q. , L. Y. He , W. H. Su , et al. 2013. “Regeneration, Recovery and Succession of a *Pinus Yunnanensis* Community Five Years After a Mega‐Fire in Central Yunnan, China.” Forest Ecology and Management 294: 188–196. 10.1016/j.foreco.2012.07.019.

[ece372911-bib-0150] Tang, C. Q. , T. Matsui , H. Ohashi , et al. 2018. “Identifying Long‐Term Stable Refugia for Relict Plant Species in East Asia.” Nature Communications 9: 4488. 10.1038/s41467-018-06837-3. PMC6203703 30367062

[ece372911-bib-0151] Tang, C. Q. , L. Q. Shen , P. B. Han , et al. 2020. “Forest Characteristics, Population Structure and Growth Trends of *Pinus Yunnanensis* in Tianchi National Nature Reserve of Yunnan, Southwestern China.” Vegetation Classification and Survey 1: 7–20. 10.3897/VCS/2020/37980.

[ece372911-bib-0152] Tilman, D. 1994. “Competition and Biodiversity in Spatially Structured Habitats.” Ecology 75: 2–16. 10.2307/1939377.

[ece372911-bib-0153] Tilman, D. , P. B. Reich , and J. M. Knops . 2006. “Biodiversity and Ecosystem Stability in a Decade‐Long Grassland Experiment.” Nature 441: 629–632. 10.1038/nature04742. 16738658

[ece372911-bib-0154] Tudor, C. , C. Constandache , L. Dinca , et al. 2025. “Pine Afforestation on Degraded Lands: A Global Review of Carbon Sequestration Potential.” Frontiers in Forests and Global Change 8: 1648094. 10.3389/ffgc.2025.1648094.

[ece372911-bib-0155] Turktas, M. , M. Aslay , E. Kaya , and F. Ertuğrul . 2012. “Molecular Characterization of Phylogenetic Relationships in *Fritillaria* Species Inferred From Chloroplast trnL‐trnF Sequences.” Turkish Journal of Biology 36: 552–560. 10.3906/biy-1201-30.

[ece372911-bib-0156] van der Sande, M. T. , L. Poorter , G. Derroire , et al. 2024. “Tropical Forest Succession Increases Tree Taxonomic and Functional Richness but Decreases Evenness.” Global Ecology and Biogeography 33: e13856. 10.1111/geb.13856.

[ece372911-bib-0157] Vellend, M. , L. Baeten , I. H. Myers‐Smith , et al. 2013. “Global Meta‐Analysis Reveals no Net Change in Local‐Scale Plant Biodiversity Over Time.” Proceedings of the National Academy of Sciences of the United States of America 110: 19456–19459. 10.1073/pnas.1312779110. 24167259 PMC3845118

[ece372911-bib-0158] Verdú, M. , P. J. Rey , J. M. Alcántara , G. Siles , and A. Valiente‐Banuet . 2009. “Phylogenetic Signatures of Facilitation and Competition in Successional Communities.” Journal of Ecology 97: 1171–1180. 10.1111/j.1365-2745.2009.01565.x.

[ece372911-bib-0159] Viana, D. S. , B. Cid , J. Figuerola , and L. Santamaría . 2016. “Disentangling the Roles of Diversity Resistance and Priority Effects in Community Assembly.” Oecologia 182: 865–875. 10.1007/s00442-016-3715-1. 27576552

[ece372911-bib-0160] Virgilio, L. R. , W. P. Ramalho , M. S. Suçuarana , and L. J. S. Vieira . 2022. “Effects of Hydrological, Environmental and Spatial Factors on Fish Diversity and Community Structure in Oxbow Lakes From the Amazon Floodplain.” Limnologica 93: 125954. 10.1016/j.limno.2022.125954.

[ece372911-bib-0161] Walker, L. R. , and R. Del Moral . 2003. Primary Succession and Ecosystem Rehabilitation. Cambridge University Press.

[ece372911-bib-0162] Walker, L. R. , D. A. Wardle , R. D. Bardgett , and B. D. Clarkson . 2010. “The Use of Chronosequences in Studies of Ecological Succession and Soil Development.” Journal of Ecology 98: 725–736. 10.1111/j.1365-2745.2010.01664.x.

[ece372911-bib-0163] Wang, G. H. , J. Y. Fang , K. Guo , et al. 2020. “Contents and Protocols for the Classification and Description of Vegetation Formations, Alliances and Associations of Vegetation of China.” Chinese Journal of Plant Ecology 44: 128–178. 10.17521/cjpe.2019.0272.

[ece372911-bib-0164] Wang, S. Y. , G. H. Lv , L. M. Jiang , H. F. Wang , Y. Li , and J. L. Wang . 2020. “Multi‐Scale Analysis on Functional Diversity and Phylogenetic Diversity of Typical Plant Community in Ebinur Lake.” Ecology and Environmental Sciences 29: 889–900. 10.16258/j.cnki.1674-5906.2020.05.004.

[ece372911-bib-0165] Wang, T. , L. Y. Xin , T. S. Ma , Z. H. Zhu , and Y. G. Du . 2022. “Effect of Phylogenetic Diversity on the Stability of Alpine Meadows on the Tibetan Plateau.” Chinese Journal of Ecology 41: 2368–2373. 10.13292/j.1000-4890.202211.019.

[ece372911-bib-0166] Warming, E. 1895. “Plantesamfund: grundtræk af den økologiske plantegeografi.” In Oecology of Plants. An Introduction to the Study of Plant‐Communities (1909). Clarendon Press.

[ece372911-bib-0168] Webb, C. O. 2000. “Exploring the Phylogenetic Structure of Ecological Communities: An Example for Rain Forest Trees.” American Naturalist 156: 145–155. 10.1086/303378. 10856198

[ece372911-bib-0167] Webb, C. O. , D. D. Ackerly , M. A. McPeek , and M. J. Donoghue . 2002. “Phylogenies and Community Ecology.” Annual Review of Ecology and Systematics 33: 475–505. 10.1146/annurev.ecolsys.33.010802.150448.

[ece372911-bib-0169] Wei, F. Y. , T. T. Xie , C. X. Su , et al. 2024. “Stability and Assembly Mechanisms of Butterfly Communities Across Environmental Gradients of a Subtropical Mountain.” Insects 15: 230. 10.3390/insects15040230. 38667360 PMC11050375

[ece372911-bib-0170] Weiher, E. , D. Freund , T. Bunton , A. Stefanski , T. Lee , and S. Bentivenga . 2011. “Advances, Challenges and a Developing Synthesis of Ecological Community Assembly Theory.” Philosophical Transactions of the Royal Society B: Biological Sciences 366: 2403–2413. 10.1098/rstb.2011.0056. PMC3130429 21768155

[ece372911-bib-0171] Wen, Q. Z. , X. S. Yang , Z. X. Yang , X. Chen , X. Lai , and F. Ding . 2010. “Dynamic Changes in *Pinus kesiya* Var. *Langbianensis* Forest Resources in China.” Resources Science 32: 1621–1626. 10.1631/jzus.A0900773.

[ece372911-bib-0172] Whitfeld, T. J. S. , W. J. Kress , D. L. Erickson , and G. D. Weiblen . 2012. “Change in Community Phylogenetic Structure During Tropical Forest Succession: Evidence From New Guinea.” Ecography 35: 821–830. 10.1111/j.1600-0587.2011.07181.x.

[ece372911-bib-0173] Whittaker, R. H. 1975. Communities and Ecosystems. 2nd ed. MacMillan.

[ece372911-bib-0174] Wiens, J. J. , D. D. Ackerly , A. P. Allen , et al. 2010. “Niche Conservatism as an Emerging Principle in Ecology and Conservation Biology.” Ecology Letters 13: 1310–1324. 10.1111/j.1461-0248.2010.01515.x. 20649638

[ece372911-bib-0175] Wu, N. C. , S. C. Zhou , M. Zhang , et al. 2021. “Spatial and Local Environmental Factors Outweigh Geo‐Climatic Gradients in Structuring Taxonomically and Trait‐Based β‐Diversity of Benthic Algae.” Journal of Biogeography 48: 1842–1857. 10.1111/jbi.14108.

[ece372911-bib-0176] Wu, Z. L. , and C. L. Dang . 1992. “The Net Primary Productivity of *Pinus kesiya* Var. *langbianensis* Stands in Pu'Er District, Yunnan.” Journal of Yunnan University 14: 128–136.

[ece372911-bib-0177] Wu, Z. Y. , and Y. C. Zhu . 1987. Yunnan vegetation. Science Press.

[ece372911-bib-0178] Xing, Y. W. , Y. S. Liu , T. Su , F. M. B. Jacques , and Z. K. Zhou . 2010. “ *Pinus prekesiya* sp. Nov. From the Upper Miocene of Yunnan, Southwestern China and Its Biogeographical Implications.” Review of Palaeobotany and Palynology 160: 1–9. 10.1016/j.revpalbo.2009.12.008.

[ece372911-bib-0179] Xu, J. S. , H. Dang , T. T. Tian , et al. 2020. “Human Disturbance Rather Than Habitat Factors Drives Plant Community Assembly and Diversity Patterns in a Semiarid Region.” Land Degradation and Development 31: 1803–1811. 10.1002/ldr.3573.

[ece372911-bib-0180] Xun, W. B. , Y. P. Liu , W. Li , et al. 2021. “Specialized Metabolic Functions of Keystone Taxa Sustain Soil Microbiome Stability.” Microbiome 9: 1–35. 10.1186/s40168-020-00985-9. 33517892 PMC7849160

[ece372911-bib-0181] Yang, Y. G. 1990. Comprehensive Physical Regionalization in Yunnan. China Higher Education Press.

[ece372911-bib-0182] Yu, Q. S. , X. Q. Rao , S. N. Ouyang , et al. 2019. “Changes in Taxonomic and Phylogenetic Dissimilarity Among Four Subtropical Forest Communities During 30 Years of Restoration.” Forest Ecology and Management 432: 983–990. 10.1016/j.foreco.2018.10.033.

[ece372911-bib-0183] Yuan, J. F. , R. Y. Hu , J. H. Shen , L. Zhang , X. Y. Zhang , and M. J. Yu . 2011. “Comparison of Species Composition and Diversity of Four Successional Forest Communities in Zhejiang Province, East China.” Bulletin of Botanical Research 31: 61–66. 10.7525/j.issn.1673-5102.2011.01.011.

[ece372911-bib-0184] Yuan, X. Q. , Z. L. Guo , S. C. Wang , et al. 2023. “Drivers and Mechanisms of Spontaneous Plant Community Succession in Abandoned PbZn Mining Areas in Yunnan, China.” Science of the Total Environment 904: 166871. 10.1016/j.scitotenv.2023.166871. 37683844

[ece372911-bib-0185] Yuan, Z. Q. , S. P. Wang , A. Gazol , et al. 2016. “Multiple Metrics of Diversity Have Different Effects on Temperate Forest Functioning Over Succession.” Oecologia 182: 1175–1185. 10.1007/s00442-016-3737-8. 27677471

[ece372911-bib-0186] Zhang, J. , S. J. Mayor , and F. He . 2014. “Does Disturbance Regime Change Community Assembly of Angiosperm Plant Communities in the Boreal Forest?” Journal of Plant Ecology 7: 188–201. 10.1093/jpe/rtt068.

[ece372911-bib-0187] Zhang, J. L. , B. Liu , S. Liu , Z. H. Feng , and K. W. Jiang . 2019. “Plantlist: Looking up the Status of Plant Scientific Names Based on the Plant List Database, Searching the Chinese Names and Making Checklists of Plants.” R package version (0.8.0). https://github.com/helixcn/plantlist.

[ece372911-bib-0188] Zhang, X. F. , D. J. He , Y. Li , et al. 2021. “Surface Fuel Loading of *Pinus massoniana* Forest in Different Succession Stages and Relevant Affecting Factors.” Forest Research 34: 108–117.

[ece372911-bib-0189] Zhang, Y. , H. Y. Chen , and P. B. Reich . 2012. “Forest Productivity Increases With Evenness, Species Richness and Trait Variation: A Global Meta‐Analysis.” Journal of Ecology 100: 742–749. 10.1111/j.1365-2745.2011.01944.x.

[ece372911-bib-0190] Zhao, Y. J. , Y. Cao , J. Wang , and Z. Xiong . 2018. “Transcriptome Sequencing of *Pinus kesiya* Var. *langbianensis* and Comparative Analysis in the *Pinus* Phylogeny.” BMC Genomics 19: 1–12. 10.1186/s12864-018-5127-6. 30285615 PMC6171231

[ece372911-bib-0191] Zhou, J. , J. J. Du , L. Bonifácio , et al. 2024. “Vulnerability of Global Pine Forestry's Carbon Sink to an Invasive Pathogen–Vector System.” Global Change Biology 30: e17614. 10.1111/gcb.17614. 39641174

