# Supplementary material for: Synchronous and Asynchronous Variation of Taxonomic and Phylogenetic Diversity During the Succession of Pinus kesiya var. langbianensis Forest in Yunnan, China
Source: Ecol Evol. 2026 Jan 12;16(1):e72911. doi: 10.1002/ece3.72911 (PMC12793900; doi:10.1002/ece3.72911)
Supplement: Supplementary file 1 — Figure S1: Relationships between field‐recorded disturbance levels and remote‐sensing‐based disturbance indicators. Figure S2: Correlation between environmental factors and spatial factors. Figure S3: Phylogenetic tree of all species in Pinus kesiya var. langbianensis forest (PKF). Figure S4: Stand structural characteristics of PKF across five successional stages. Figure S5: Variation trend of explanatory rate of spatial factors and environmental factors in PKF succession processes. Table S1: Field investigation plots of Pinus kesiya var. langbianensis forest (PKF). Table S2: 16 MEM principal component axes with significant positive eigenvalues. Table S3: List of screened explanatory variables. Table S4: Results of DCA ordination parameters. Table S5: Mantel test of taxonomic β diversity (TβD) and phylogenetic β diversity (PβD) at different successional stages of PKF. [file ECE3-16-e72911-s002.docx]

# Supplementary Information

**Synchronous and asynchronous variation of taxonomic and phylogenetic diversity during the succession of *Pinus kesiya* var. *langbianensis* forest in Yunnan, China**

**Fig. S1.** Relationships between field-recorded disturbance levels and remote-sensing-based disturbance indicators.

**Fig. S2.** Correlation between environmental factors and spatial factors.

**Fig. S3.** Phylogenetic tree of all species in *Pinus kesiya* var. *langbianensis* forest (PKF).

**Fig. S4.** Stand structural characteristics of PKF across five successional stages.

**Fig. S5.** Variation trend of explanatory rate of spatial factors and environmental factors in PKF succession processes.

**Table S1.** Field investigation plots of *Pinus kesiya* var. *langbianensis* forest (PKF).

**Table S2.** 16 MEM principal component axes with significant positive eigenvalues.

**Table S3.** List of screened explanatory variables.

**Table S4.** Results of DCA ordination parameters.

**Table S5.** Mantel test of taxonomic β diversity (TβD) and phylogenetic β diversity (PβD) at different successional stages of PKF.





## **Fig. S1**. Relationships between field-recorded disturbance levels and remote-sensing-based disturbance indicators. (a): Field recorded human disturbance levels vs Human Footprint Index; (b): Field recorded wildfire levels vs MODIS-derived burned area.


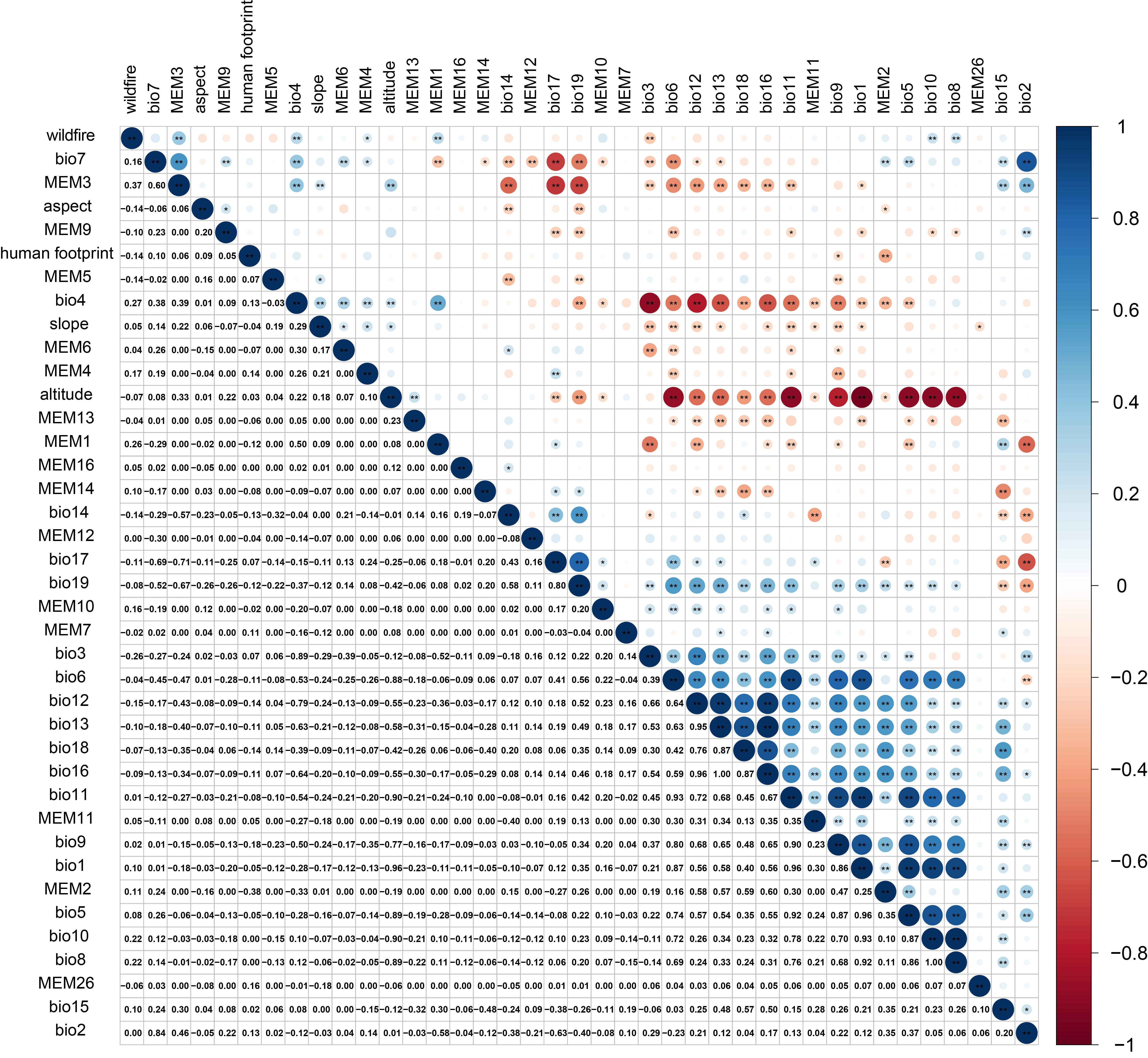


## **Fig. S2**. Correlation between environmental factors and spatial factors.


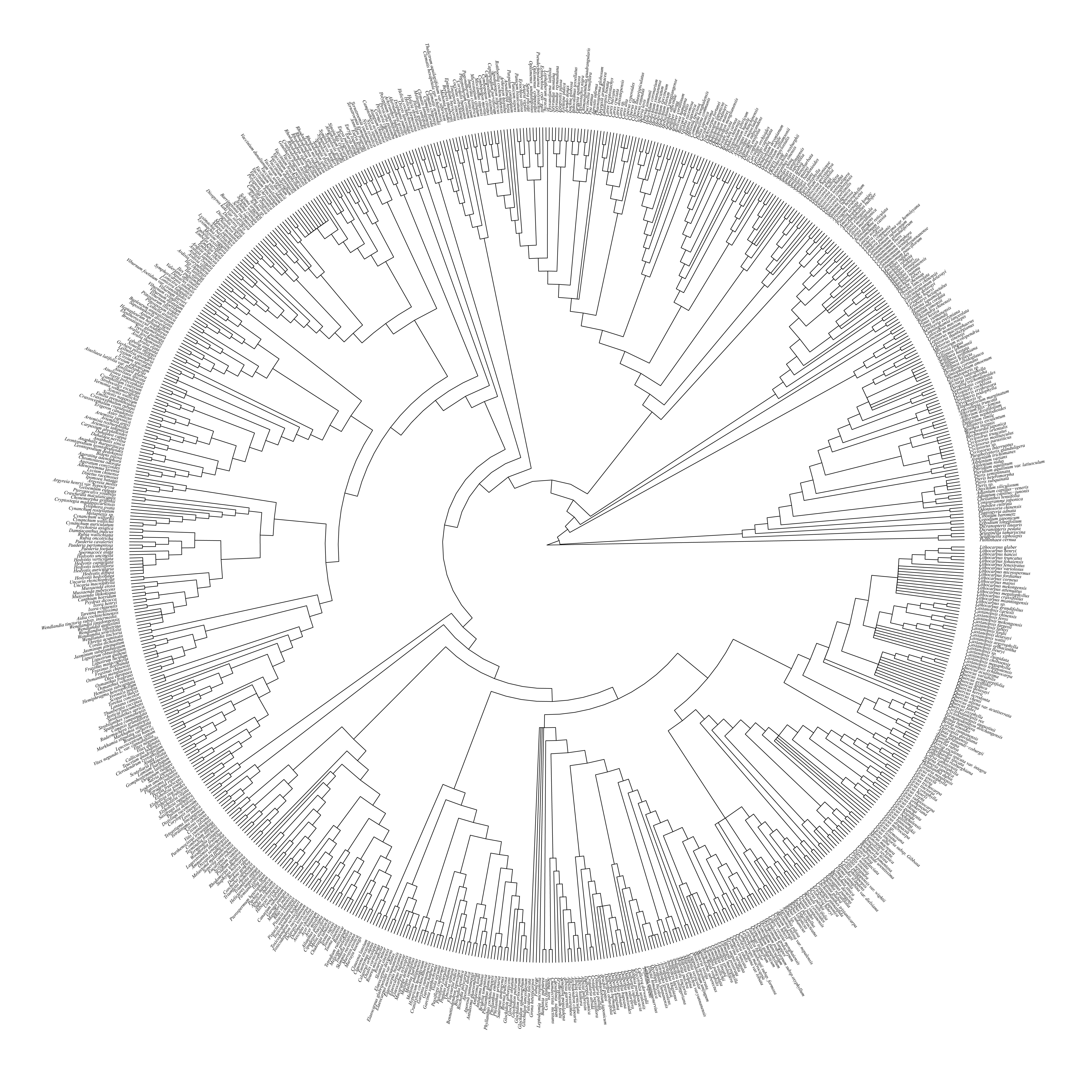


## **Fig. S3.** Phylogenetic tree of all species in *Pinus kesiya* var. *langbianensis* forest (PKF).





## **Fig. S4.** Stand structural characteristics of PKF across five successional stages. (a) Tree density; (b) Tree cover; (c) Stand height.


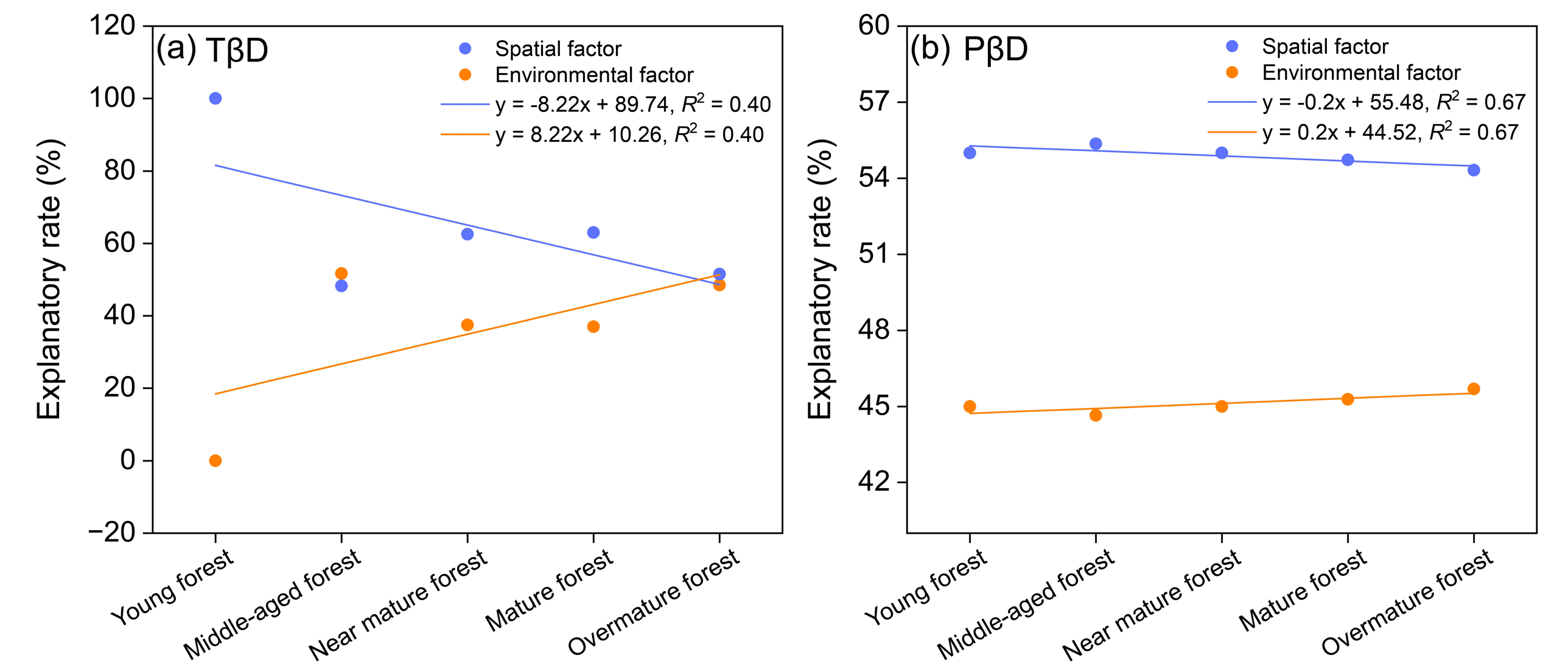


## **Fig. S5.** Variation trend of explanatory rate of spatial factors and environmental factors in PKF succession processes. TβD - taxonomic β diversity; PβD - phylogenetic β diversity.

## **Table S1.** Field investigation plots of *Pinus* *kesiya* var. *langbianensis* forest (PKF).

| Plots | Longitude / ° | Latitude / ° | Age group | Age class stage  (a) | Tree density (ind/ha) | Tree cover (%) | Stand height (m) |
| --- | --- | --- | --- | --- | --- | --- | --- |
| S1 | 100.91899 | 24.46616 | Young forest | ≤20 | 2333.33 | 80 | 12.20 |
| S2 | 100.86148 | 24.61842 | Near mature forest | 31–40 | 2016.67 | 80 | 18.00 |
| S3 | 100.74147 | 24.48835 | Near mature forest | 31–40 | 966.67 | 85 | 12.30 |
| S4 | 100.47595 | 24.66513 | Middle-aged forest | 21-30 | 1050.00 | 90 | 14.50 |
| S5 | 100.53846 | 24.54493 | Young forest | ≤20 | 1433.33 | 87 | 6.60 |
| S6 | 100.86008 | 24.35641 | Middle-aged forest | 21–30 | 2950.00 | 90 | 5.90 |
| S7 | 100.63581 | 24.33519 | Young forest | ≤20 | 2100.00 | 80 | 12.00 |
| S8 | 100.97862 | 24.01318 | Mature forest | 41–60 | 2066.67 | 80 | 18.30 |
| S9 | 101.13298 | 24.02787 | Middle-aged forest | 21–30 | 2850.00 | 75 | 9.80 |
| S10 | 100.61778 | 23.75223 | Young forest | ≤20 | 2466.67 | 79 | 13.70 |
| S11 | 100.68991 | 23.98888 | Middle-aged forest | 21–30 | 1916.67 | 86 | 9.00 |
| S12 | 100.79834 | 24.02871 | Young forest | ≤20 | 1633.33 | 75 | 14.20 |
| S13 | 100.51401 | 23.19271 | Young forest | ≤20 | 2216.67 | 85 | 13.60 |
| S14 | 100.50679 | 23.23967 | Young forest | ≤20 | 3500.00 | 70 | 12.20 |
| S15 | 100.51768 | 23.32529 | Near mature forest | 31–40 | 3416.67 | 65 | 10.00 |
| S16 | 100.46804 | 23.15360 | Middle-aged forest | 21–30 | 2466.67 | 75 | 9.70 |
| S17 | 100.45973 | 23.09888 | Young forest | ≤20 | 3316.67 | 70 | 13.00 |
| S18 | 100.71469 | 22.90763 | Near mature forest | 31–40 | 2716.67 | 75 | 18.80 |
| S19 | 100.70184 | 22.98477 | Middle-aged forest | 21–30 | 2500.00 | 76 | 11.00 |
| S20 | 100.97390 | 22.72163 | Young forest | ≤20 | 2566.67 | 85 | 8.90 |
| S21 | 100.91268 | 22.71988 | Middle-aged forest | 21–30 | 3600.00 | 80 | 10.80 |
| S22 | 100.90955 | 22.38350 | Mature forest | 41–60 | 1100.00 | 80 | 21.00 |
| S23 | 101.61976 | 23.42541 | Near mature forest | 31–40 | 2116.67 | 65 | 20.00 |
| S24 | 101.59039 | 23.37531 | Middle-aged forest | 21–30 | 2266.67 | 70 | 16.00 |
| S25 | 101.46828 | 23.31938 | Mature forest | 41–60 | 2083.33 | 60 | 23.00 |
| S26 | 101.41537 | 23.29139 | Middle-aged forest | 21–30 | 2566.67 | 80 | 19.00 |
| S27 | 101.46931 | 23.18632 | Near mature forest | 31–40 | 2866.67 | 85 | 35.00 |
| S28 | 101.45833 | 23.46513 | Middle-aged forest | 21–30 | 1850.00 | 60 | 12.00 |
| S29 | 101.30121 | 23.62311 | Near mature forest | 31–40 | 2050.00 | 80 | 26.00 |
| S30 | 101.24104 | 23.76387 | Mature forest | 41–60 | 2850.00 | 75 | 33.00 |
| S31 | 101.27568 | 23.93816 | Overmature forest | ≥61 | 2316.67 | 65 | 36.00 |
| S32 | 101.13872 | 23.94225 | Mature forest | 41–60 | 1750.00 | 70 | 30.00 |
| S33 | 100.94370 | 23.93000 | Overmature forest | ≥61 | 1816.67 | 50 | 17.00 |
| S34 | 100.78586 | 23.65924 | Overmature forest | ≥61 | 2516.67 | 60 | 30.00 |
| S35 | 100.83176 | 23.43803 | Mature forest | 41–60 | 2150.00 | 65 | 20.00 |
| S36 | 100.87853 | 23.21515 | Middle-aged forest | 21–30 | 3383.33 | 90 | 22.00 |
| S37 | 101.03652 | 23.10052 | Overmature forest | ≥61 | 1783.33 | 50 | 32.00 |
| S38 | 101.13597 | 23.21286 | Near mature forest | 31–40 | 1450.00 | 60 | 18.00 |
| S39 | 101.13419 | 23.40975 | Near mature forest | 31–40 | 3266.67 | 85 | 22.50 |
| S40 | 101.15465 | 23.31141 | Middle-aged forest | 21–30 | 1983.33 | 75 | 18.00 |
| S41 | 101.14958 | 23.08100 | Near mature forest | 31–40 | 2300.00 | 80 | 26.00 |
| S42 | 101.23207 | 22.96751 | Near mature forest | 31–40 | 2283.33 | 80 | 27.00 |
| S43 | 101.45528 | 22.79342 | Middle-aged forest | 21–30 | 2766.67 | 85 | 23.00 |
| S44 | 101.65590 | 22.68637 | Middle-aged forest | 21–30 | 1933.33 | 80 | 23.00 |
| S45 | 101.85237 | 22.60332 | Near mature forest | 31–40 | 2466.67 | 90 | 22.00 |
| S46 | 101.90727 | 22.74140 | Mature forest | 41–60 | 1750.00 | 80 | 23.00 |
| S47 | 101.87857 | 22.75394 | Middle-aged forest | 21–30 | 2066.67 | 85 | 22.00 |
| S48 | 101.78598 | 22.58864 | Overmature forest | ≥61 | 1783.33 | 70 | 32.00 |
| S49 | 101.43580 | 22.62465 | Mature forest | 41–60 | 3000.00 | 80 | 23.60 |
| S50 | 101.37850 | 22.68862 | Near mature forest | 31–40 | 2516.67 | 90 | 22.00 |
| S51 | 101.28929 | 22.74707 | Near mature forest | 31–40 | 1366.67 | 70 | 20.00 |
| S52 | 101.16291 | 22.71310 | Mature forest | 41–60 | 4450.00 | 85 | 65.00 |
| S53 | 100.92559 | 22.88753 | Mature forest | 41–60 | 2850.00 | 80 | 23.00 |
| S54 | 100.75060 | 22.65820 | Near mature forest | 31–40 | 2433.33 | 65 | 22.00 |
| S55 | 100.36805 | 22.51580 | Overmature forest | ≥61 | 2833.33 | 70 | 22.00 |
| S56 | 99.92614 | 22.59727 | Middle-aged forest | 21–30 | 2716.67 | 85 | 18.00 |
| S57 | 100.10923 | 22.59723 | Middle-aged forest | 21–30 | 2566.67 | 75 | 22.60 |
| S58 | 100.14382 | 22.65989 | Middle-aged forest | 21–30 | 2450.00 | 80 | 25.00 |
| S59 | 100.17917 | 22.74036 | Mature forest | 41–60 | 1800.00 | 75 | 22.70 |
| S60 | 99.86835 | 22.56331 | Near mature forest | 31–40 | 2266.67 | 75 | 20.00 |
| S61 | 99.67332 | 22.62321 | Middle-aged forest | 21–30 | 3133.33 | 90 | 17.00 |
| S62 | 99.64020 | 22.60371 | Mature forest | 41–60 | 2250.00 | 60 | 25.00 |
| S63 | 99.62216 | 22.54966 | Middle-aged forest | 21–30 | 1483.33 | 90 | 20.00 |
| S64 | 99.66878 | 22.46955 | Mature forest | 41–60 | 2583.33 | 85 | 25.00 |
| S65 | 99.42576 | 22.21611 | Mature forest | 41–60 | 2633.33 | 75 | 20.00 |
| S66 | 99.66356 | 22.33074 | Mature forest | 41–60 | 2800.00 | 85 | 23.00 |
| S67 | 99.62375 | 24.78217 | Near mature forest | 31–40 | 2500.00 | 80 | 22.50 |
| S68 | 99.70848 | 24.90704 | Near mature forest | 31–40 | 2066.67 | 65 | 22.50 |
| S69 | 99.53889 | 24.78093 | Mature forest | 41–60 | 1833.33 | 60 | 18.90 |
| S70 | 99.35134 | 24.75485 | Overmature forest | ≥61 | 1866.67 | 75 | 25.50 |
| S71 | 99.50451 | 24.73394 | Mature forest | 41–60 | 2016.67 | 65 | 23.20 |
| S72 | 100.43889 | 22.31716 | Overmature forest | ≥61 | 1566.67 | 60 | 20.20 |
| S73 | 100.41160 | 22.30684 | Mature forest | 41–60 | 1650.00 | 60 | 16.00 |
| S74 | 100.39675 | 22.27772 | Middle-aged forest | 21–30 | 1950.00 | 50 | 13.80 |
| S75 | 100.50563 | 22.25213 | Overmature forest | ≥61 | 1366.67 | 60 | 23.50 |
| S76 | 100.44179 | 22.26937 | Mature forest | 41–60 | 1483.33 | 65 | 28.00 |
| S77 | 100.42413 | 22.16591 | Near mature forest | 31–40 | 2150.00 | 60 | 21.80 |
| S78 | 100.39047 | 22.16729 | Overmature forest | ≥61 | 1633.33 | 60 | 20.50 |
| S79 | 100.33717 | 22.18327 | Young forest | ≤20 | 1016.67 | 80 | 15.60 |
| S80 | 100.33281 | 22.09651 | Mature forest | 41–60 | 2250.00 | 65 | 23.90 |
| S81 | 100.39000 | 22.03694 | Mature forest | 41–60 | 1583.33 | 90 | 29.50 |
| S82 | 101.69722 | 21.23240 | Overmature forest | ≥61 | 1750.00 | 80 | 28.90 |
| S83 | 100.00347 | 22.14292 | Mature forest | 41–60 | 1200.00 | 75 | 31.40 |
| S84 | 100.01523 | 22.16172 | Mature forest | 41–60 | 1633.33 | 90 | 22.60 |
| S85 | 100.00936 | 22.03329 | Near mature forest | 31–40 | 3050.00 | 60 | 21.70 |
| S86 | 99.83591 | 22.09288 | Overmature forest | ≥61 | 2033.33 | 65 | 25.30 |
| S87 | 99.82959 | 22.11743 | Mature forest | 41–60 | 1816.67 | 85 | 31.70 |
| S88 | 99.75965 | 22.13426 | Mature forest | 41–60 | 2500.00 | 75 | 25.30 |
| S89 | 99.77361 | 22.21046 | Middle-aged forest | 21–30 | 1933.33 | 65 | 27.30 |
| S90 | 100.17560 | 21.91958 | Overmature forest | ≥61 | 1900.00 | 70 | 28.90 |
| S91 | 100.35404 | 21.78006 | Young forest | ≤20 | 3733.33 | 80 | 17.10 |
| S92 | 101.46416 | 21.99031 | Mature forest | 41–60 | 1850.00 | 85 | 29.70 |
| S93 | 101.52248 | 22.23475 | Overmature forest | ≥61 | 1633.33 | 75 | 33.50 |
| S94 | 101.67111 | 21.25058 | Overmature forest | ≥61 | 1783.33 | 55 | 26.40 |
| S95 | 101.70472 | 21.22972 | Overmature forest | ≥61 | 1683.33 | 70 | 30.20 |
| S96 | 100.92133 | 22.38908 | Near mature forest | 31–40 | 1416.67 | 65 | 27.70 |
| S97 | 100.97413 | 22.34693 | Mature forest | 41–60 | 1783.33 | 60 | 29.50 |
| S98 | 101.02730 | 22.33219 | Mature forest | 41–60 | 1566.67 | 85 | 34.10 |
| S99 | 100.99311 | 22.41589 | Overmature forest | ≥61 | 1933.33 | 80 | 31.50 |
| S100 | 101.10043 | 22.41991 | Overmature forest | ≥61 | 1766.67 | 85 | 24.40 |
| S101 | 100.85880 | 22.07783 | Young forest | ≤20 | 3466.67 | 85 | 17.30 |
| S102 | 100.16128 | 22.13123 | Near mature forest | 31–40 | 1633.33 | 50 | 19.20 |
| S103 | 99.64630 | 22.19794 | Middle-aged forest | 21–30 | 2416.67 | 80 | 15.00 |
| S104 | 99.61155 | 22.17573 | Middle-aged forest | 21–30 | 1616.67 | 60 | 22.70 |
| S105 | 99.69969 | 22.17440 | Near mature forest | 31–40 | 2100.00 | 65 | 36.50 |
| S106 | 100.66961 | 23.59016 | Mature forest | 41–60 | 1933.33 | 70 | 18.00 |
| S107 | 100.57403 | 23.50189 | Near mature forest | 31–40 | 1533.33 | 55 | 18.00 |
| S108 | 100.62354 | 23.34206 | Mature forest | 41–60 | 1950.00 | 65 | 16.80 |
| S109 | 100.57462 | 23.22620 | Overmature forest | ≥61 | 1583.33 | 60 | 32.00 |
| S110 | 100.57486 | 23.14645 | Overmature forest | ≥61 | 1300.00 | 60 | 20.00 |
| S111 | 100.62428 | 23.28230 | Near mature forest | 31–40 | 1783.33 | 55 | 30.00 |
| S112 | 100.55673 | 23.19459 | Mature forest | 41–60 | 2033.33 | 60 | 27.00 |
| S113 | 100.55913 | 23.19435 | Mature forest | 41–60 | 2483.33 | 70 | 33.00 |
| S114 | 100.56230 | 23.19391 | Near mature forest | 31–40 | 1200.00 | 90 | 27.80 |
| S115 | 100.56477 | 23.19504 | Overmature forest | ≥61 | 1400.00 | 50 | 27.80 |
| S116 | 100.56847 | 23.19417 | Overmature forest | ≥61 | 1600.00 | 55 | 28.00 |
| S117 | 100.54411 | 23.13123 | Overmature forest | ≥61 | 1650.00 | 60 | 35.50 |
| S118 | 100.54550 | 23.13115 | Near mature forest | 31–40 | 2083.33 | 60 | 27.60 |
| S119 | 100.14766 | 23.13031 | Mature forest | 41–60 | 1383.33 | 55 | 40.00 |
| S120 | 100.55258 | 23.12682 | Overmature forest | ≥61 | 1983.33 | 60 | 34.30 |
| S121 | 100.55649 | 23.12450 | Mature forest | 41–60 | 1683.33 | 55 | 26.00 |
| S122 | 100.59965 | 23.30602 | Mature forest | 41–60 | 2100.00 | 70 | 24.80 |
| S123 | 100.57226 | 23.29140 | Near mature forest | 31–40 | 2233.33 | 70 | 27.30 |
| S124 | 100.35976 | 23.58225 | Near mature forest | 31–40 | 1500.00 | 50 | 21.00 |
| S125 | 100.15375 | 23.52451 | Near mature forest | 31–40 | 2133.33 | 65 | 34.00 |
| S126 | 100.11688 | 23.58425 | Near mature forest | 31–40 | 1816.67 | 70 | 22.00 |
| S127 | 100.10078 | 23.94298 | Middle-aged forest | 21–30 | 1683.33 | 50 | 10.40 |
| S128 | 100.16295 | 23.90322 | Near mature forest | 31–40 | 1016.67 | 75 | 23.00 |
| S129 | 99.96510 | 23.06070 | Mature forest | 41–60 | 1900.00 | 55 | 21.50 |
| S130 | 99.65424 | 23.46943 | Middle-aged forest | 21–30 | 1583.33 | 50 | 14.30 |
| S131 | 99.71999 | 23.48132 | Mature forest | 41–60 | 1583.33 | 60 | 25.80 |
| S132 | 99.75060 | 23.48854 | Mature forest | 41–60 | 1683.33 | 50 | 18.00 |
| S133 | 99.77561 | 23.36850 | Near mature forest | 31–40 | 1783.33 | 60 | 19.00 |
| S134 | 99.72534 | 22.09698 | Overmature forest | ≥61 | 2100.00 | 65 | 36.50 |
| S135 | 99.30164 | 24.18791 | Near mature forest | 31–40 | 1100.00 | 60 | 15.20 |
| S136 | 99.3145 | 24.58312 | Near mature forest | 31–40 | 2250.00 | 55 | 18.00 |
| S137 | 99.1654 | 24.11056 | Near mature forest | 31–40 | 1400.00 | 50 | 16.00 |
| S138 | 99.01127 | 24.308731 | Overmature forest | ≥61 | 1566.67 | 60 | 19.00 |
| S139 | 99.2279 | 24.12396 | Near mature forest | 31–40 | 2316.67 | 50 | 18.00 |
| S140 | 98.46818 | 24.43145 | Overmature forest | ≥61 | 1316.67 | 45 | 14.80 |
| S141 | 98.25662 | 24.51272 | Mature forest | 41–60 | 933.33 | 45 | 18.40 |
| S142 | 99.2095 | 24.708729 | Mature forest | 41–60 | 1400.00 | 50 | 19.80 |
| S143 | 101.375173 | 23.935864 | Mature forest | 41–60 | 1933.33 | 45 | 40.00 |
| S144 | 101.355248 | 23.935136 | Middle-aged forest | 21–30 | 1333.33 | 60 | 16.00 |

**Reference:**

National Forestry and Grassland Administration, 2017. People’s Republic of China (PRC) Forestry Industry Standard. Regulations for age-class and age-group division of main tree-species. LY/T 2908–2017.

| Variables | Order | *F* | *p*-value |
| --- | --- | --- | --- |
| MEM5 | 5 | 2.858146 | 0.001 |
| MEM2 | 2 | 2.852696 | 0.001 |
| MEM1 | 1 | 2.042408 | 0.001 |
| MEM6 | 6 | 2.050592 | 0.001 |
| MEM4 | 4 | 1.795307 | 0.001 |
| MEM14 | 14 | 1.794541 | 0.001 |
| MEM3 | 3 | 1.671339 | 0.001 |
| MEM7 | 7 | 1.670075 | 0.001 |
| MEM9 | 9 | 1.530198 | 0.005 |
| MEM11 | 11 | 1.522907 | 0.004 |
| MEM13 | 13 | 1.498376 | 0.007 |
| MEM12 | 12 | 1.39269 | 0.008 |
| MEM26 | 26 | 1.319188 | 0.022 |
| MEM16 | 16 | 1.300404 | 0.035 |
| MEM10 | 10 | 1.294232 | 0.029 |
| MEM19 | 19 | 1.291195 | 0.037 |

## **Table S2.** 16 MEM principal component axes with significant positive eigenvalues.

## **Table S3.** List of screened explanatory variables.

| Type | Variables | VIF |
| --- | --- | --- |
| Environment factors | **altitude (m)*** | **1.384261** |
|  | **slope (°)** | **1.454768** |
|  | aspect (°) | 169.3977 |
|  | bio1 (Annual mean temperature / °C) | 16085.483645 |
|  | bio2 (Mean diurnal range / °C) | 13096.692814 |
|  | **bio3 (Isothermality / -)** | **4.954630** |
|  | bio4 (Temperature seasonality / -) | 5433.635568 |
|  | bio5 (Max temperature of warmest month / °C) | 36565.569842 |
|  | bio6 (Min temperature of coldest month / °C) | 45626.856325 |
|  | **bio7 (Temperature annual range / °C)** | **2.234895** |
|  | bio8 (Mean temperature of wettest quarter / °C) | 505.690425 |
|  | **bio9 (Mean temperature of driest quarter / °C)** | **1.119121** |
|  | bio10 (Mean temperature of warmest quarter / °C) | 5914.884226 |
|  | bio11 (Mean temperature of coldest quarter / °C) | 30166.014062 |
|  | bio12 (Annual precipitation / mm) | 1655.955120 |
|  | bio13 (Precipitation of wettest month / mm) | 425.704333 |
|  | **bio14 (Precipitation of driest month / mm)** | **4.573980** |
|  | **bio15 (Precipitation seasonality / -)** | **4.347468** |
|  | bio16 (Precipitation of wettest quarter / mm) | 1362.195706 |
|  | bio17 (Precipitation of driest quarter / mm) | 67.350225 |
|  | bio18 (Precipitation of warmest quarter / mm) | 14.370068 |
|  | **bio19 (Precipitation of coldest quarter / mm)** | **4.692419** |
|  | **human footprint** | **1.458082** |
|  | **wildfire** | **1.725434** |
| Spatial factors | **MEM1** | **2.205987** |
|  | **MEM2** | **3.386912** |
|  | **MEM3** | **4.628656** |
|  | **MEM4** | **1.686696** |
|  | **MEM5** | **2.015240** |
|  | **MEM6** | **1.965285** |
|  | **MEM7** | **1.175982** |
|  | MEM9 | 17.395761 |
|  | **MEM10** | **1.693252** |
|  | **MEM11** | **3.058971** |
|  | MEM12 | 24.942389 |
|  | **MEM13** | **1.560422** |
|  | **MEM14** | **2.472239** |
|  | **MEM16** | **1.268247** |
|  | MEM19 | 26.880534 |
|  | MEM26 | 22.542162 |

* Explanatory variables in bold are chosen for the analysis.

## **Table S4.** Results of DCA ordination parameters.

| Layer | DCA1 | DCA2 | DCA3 | DCA4 |
| --- | --- | --- | --- | --- |
| Eigenvalues | 0.4325 | 0.3509 | 0.3488 | 0.3544 |
| Decorana values | 0.4994 | 0.4413 | 0.3959 | 0.3522 |
| Axis lengths | **4.1672** | 5.2133 | 3.6518 | 3.1532 |

| Tppe | | altitude | slope | bio3 | bio7 | bio9 | bio14 | bio15 | bio19 | human footprint | wildfire |
| --- | --- | --- | --- | --- | --- | --- | --- | --- | --- | --- | --- |
| Young forest | TβD | -0.076 | 0.162 | -0.087 | 0.030 | 0.176 | 0.029 | -0.093 | -0.057 | -0.157 | -0.091 |
|  | PβD | -0.003 | 0.251 | -0.175 | -0.188 | 0.086 | -0.238 | -0.120 | -0.204 | -0.219 | -0.099 |
| Middle-aged forest | TβD | 0.089 | -0.013 | -0.045 | **0.161**** | -0.086 | 0.036 | **0.143**** | 0.035 | -0.061 | 0.008 |
|  | PβD | 0.077 | -0.017 | 0.006 | -0.009 | 0.017 | 0.085 | 0.046 | 0.117 | 0.215 | -0.043 |
| Near mature forest | TβD | 0.059 | 0.013 | 0.056 | **0.196**** | **0.194**** | 0.028 | 0.191** | -0.026 | 0.026 | 0.164** |
|  | PβD | 0.030 | 0.080 | -0.013 | 0.039 | 0.022 | -0.042 | 0.021 | -0.130 | 0.045 | 0.148 |
| Mature forest | TβD | -0.026 | -0.035 | **0.176**** | -0.007 | 0.049 | 0.047 | **0.199**** | 0.059 | 0.030 | 0.044 |
|  | PβD | 0.208 | 0.038 | 0.153 | 0.011 | **0.406**** | 0.001 | **0.337**** | -0.044 | 0.025 | -0.002 |
| Overmature forest | TβD | 0.074 | 0.144 | 0.058 | **0.202**** | **0.202**** | 0.052 | 0.197** | 0.190** | **0.437***** | 0.055 |
|  | PβD | 0.054 | 0.132 | -0.027 | 0.037 | **0.348**** | -0.104 | 0.186 | -0.003 | **0.492***** | -0.022 |

## **Table S5.** Mantel test of taxonomic β diversity (TβD) and phylogenetic β diversity (PβD) at different successional stages of PKF.

Significance level: ‘**’ *P* < 0.01, ‘*’ *P* < 0.05.
